# Metabolic regulation of behavior by the intestinal enzyme FMO-2

**DOI:** 10.1126/sciadv.adx3018

**Published:** 2025-10-24

**Authors:** Elizabeth S. Kitto, Safa Beydoun, Ella Henry, Megan L. Schaller, Mira Bhandari, Sarah A. Easow, Angela M. Tuckowski, Marshall B. Howington, Ajay Bhat, Aditya Sridhar, Eugene Chung, Charles R. Evans, Scott F. Leiser

**Affiliations:** ^1^ Molecular and Integrative Physiology Department, University of Michigan, Ann Arbor, MI, USA.; ^2^ Cellular and Molecular Biology Program, University of Michigan, Ann Arbor, MI, USA.; ^3^ Biomedical Research Core Facilities Metabolomics Core, University of Michigan, Ann Arbor, MI, USA.; ^4^ Department of Internal Medicine, University of Michigan, Ann Arbor, MI, USA.

## Abstract

Many elements of an organism’s behavior are intertwined with the organism’s health. Over a long period of time, health status is also indicative of life span, with improved health correlating with a longer life. However, the relationship between longevity and behavior remains relatively unexplored. Here, we report that modification of a single longevity gene downstream of dietary restriction and hypoxia markedly alters behavior in *Caenorhabditis elegans*. We found that modified expression of flavin-containing monooxygenase (*fmo-2*) leads to altered sensory perception and decision-making in a variety of behavioral paradigms. This cell nonautonomous signaling pathway is linked to changes in tryptophan metabolism, where loss of *fmo-2* requires the tryptophan metabolite serotonin and overexpressed *fmo-2* requires the tryptophan metabolite quinolinic acid to change behavior. These results suggest a unique mechanism for gut metabolism to communicate positive satiety signals and negative depressive signals to the organism by modifying an essential amino acid. They also demonstrate the importance of examining pleiotropic effects in promising longevity interventions.

## Teaser

The intestinal pro-longevity enzyme *fmo-2* interacts with tryptophan-derived neuromodulators to change behavior.

## INTRODUCTION

Within the past 30 years, there has been a growing interest in researching environmental and genetic factors that determine the rate of aging across taxa (*1*). The ability of environmental factors to slow aging was first reported in 1935, when McCay *et al.* (*2*) published that caloric restriction extends life span in rodents. In 1983, the first genetic mutants with increased life span were identified (*3*), showing that it is possible to identify physiological drivers of aging and to manipulate these pathways to extend life span. In the ensuing decades, many physiological circuits that modify the aging process have been identified in multiple model organisms. These interventions, including dietary restriction (DR) (*4*), temperature stress (*5*, *6*), insulin signaling (*7*–*9*), and hypoxia (*10*, *11*), share a common theme in that they activate stress response pathways without damaging the organism. This concept of mild stressors activating beneficial stress response pathways to improve health and longevity is known as hormesis (*12*). However, the ability of a stressor to induce a beneficial hormetic effect depends on the baseline health of the organism, the dose of the stressor, and levels of other, co-occurring environmental stressors (*13*).

Consequently, environmental manipulations that extend life span are often impractical solutions to improving healthspan on a population-wide scale. Interventions like DR and other stressors can have low compliance, are difficult to titrate to each individual, and elicit broad physiological changes that affect phenotypes other than aging (*14*–*17*). One potential solution to these implementation problems is to activate stress-response pathways via genetic or pharmaceutical interventions. For example, DR extends life span across taxa through multiple mechanisms including decreased insulin and mammalian target of rapamycin (mTOR) signaling (*18*). Genetic manipulations that reduce insulin and growth hormone signaling mimic DR to extend life span across taxa (*19*–*21*). Pharmaceutical interventions such as rapamycin can also mimic DR by inhibiting mTOR signaling to improve health (*22*). Now, much work is focused on identifying pro-longevity circuits downstream of environmental interventions, with the idea that targeted pharmaceutical manipulation of these pathways is a more practical solution than administering stressors like DR to human patients (*23*).

Similarly, genes acting downstream of multiple longevity interventions are of particular interest because they could be highly specific modulators of aging and may be more likely to extend life span in a diverse population. One such gene is flavin-containing monooxygenase (FMO). In *Caenorhabditis elegans*, *fmo-2* expression is required for the longevity interventions DR and hypoxia to extend life span, and overexpression of *fmo-2* is sufficient to extend life span by modifying one-carbon and tryptophan metabolism (*24*). Moreover, FMOs (i) are structurally conserved from worms to humans, (ii) are up-regulated in multiple long-lived mouse models (*25*–*27*), (iii) improve stress resistance in human cells when overexpressed (*28*), (iv) are induced by multiple Food and Drug Administration–approved pharmaceuticals (*29*), and (v) promote health span in *C. elegans* and mammalian cell culture (*29*, *30*). Together, these results make Fmos an important gene family to understand for their conserved role in health and longevity.

Pharmaceutical and genetic interventions that extend life span through a single gene (e.g., Fmo) are more practical and targeted than environmental longevity interventions. However, it remains unclear whether these strategies will also have pleiotropic effects on other areas of physiology such as reproduction, stress resistance, and behavior. For example, many longevity interventions decrease fertility by directing resources toward preserving somatic function over germline health (*31*). In addition, some of these interventions may extend life span in a highly controlled laboratory environment but reduce an organism’s ability to withstand real-world stressors like pathogens (*32*–*34*). Acute and chronic stress exposure also has profound effects on behavior and mental state (*35*, *36*). For example, attempts to restrict calories in humans can lead to decreased cognitive performance and dysphoria (*37*). Stresses induced by other longevity interventions, like heat stress (*38*) and hypoxic stress (*39*), are also linked to negative mental health outcomes in people.

Many longevity interventions extend life span by acting on key metabolic tissues like the liver (*40*) and intestine (*41*). Although the role of these tissues in neurosignaling and mental state is not well understood, there is growing evidence that these tissues directly and indirectly affect the nervous system and behavior. For example, broad metabolic changes induced by a ketogenic diet have been used since the 1920s to treat epilepsy, although the mechanism of this treatment is still not fully understood (*42*). In addition, general metabolic dysfunction—characterized by elevated circulating glucose and triglycerides—is also correlated with poor mental health outcomes (*43*). Last, a growing interest in the gut-brain axis has revealed that metabolites produced by intestinal microbes can act as neuromodulators (*44*–*46*) and alter behavior. Many canonical neurotransmitters are also synthesized in the intestine and able to cross the blood-brain barrier (*47*), pointing to an essential role for peripheral metabolism in neuronal activity. Although both longevity and behavior are heavily intertwined with metabolism, whether substantial metabolic connections between behavior and longevity exist is less well explored. Consequently, it is essential to examine potential interactions between life span and behavior in longevity interventions that target these metabolically active tissues.

In this work, we explore how the pro-longevity gene *fmo-2* modifies *C. elegans* behavior and whether these behavioral effects are separable from *fmo-2*’s role in longevity. We find that *fmo-2* overexpression leads to a variety of sensory perception and decision-making–related behaviors in *C. elegans*, such as decreased exploration of a novel environment. Our data suggest that this low-exploration phenotype results from FMO-2’s role in oxygenating tryptophan to produce *N*-formylkynurenine. Overexpressing *fmo-2* alters tryptophan flux toward the kynurenine-derived neuromodulators quinolinic acid and kynurenic acid, leading to decreased exploration. This low-exploration phenotype can be rescued genetically by blocking quinolinic acid production or signaling. We find that decreasing *fmo-2* expression through genetic knockdown or knockout also leads to a low-exploration phenotype. In contrast to *fmo-2* overexpression, we find that the behavioral effects of low *fmo-2* expression require tryptophan flux toward the neurotransmitter serotonin. These findings lead to a “goldilocks” effect, where a wild-type (WT) level of *fmo-2* expression is required for a normal flux through tryptophan metabolism and, in turn, normal exploratory behavior. Last, we find that the genetic interventions that rescue *fmo-2*–mediated behavioral effects also rescue some, but not all, of the other behavioral and aging-related phenotypes observed in these strains. Together, these results point toward a central role for *C. elegans* FMO-2 and tryptophan metabolism in modifying longevity and behavior that serves as an example for how longevity, metabolism, and behavior can be intertwined.

## RESULTS

### *fmo-2* expression alters *C. elegans* sensory perception and foraging

To investigate whether overexpressing the metabolic enzyme *fmo-2* affects “mental state,” we created two panels of behavioral assays to run on WT and *fmo-2* overexpressor (OE) animals at day 1 of adulthood. The first of these panels was designed to test locomotor ability to ensure that differences in movement capacity would not interfere with measurements of more complex behaviors tested in the second panel. In our resulting data (fig. S1A), we observed no differences in maximum velocity (fig. S1B), pumping (fig. S1C), or thrashing (fig. S1D) between WT and OE animals. These results support that *fmo-2* overexpressors have a similar ability to physically move in different environments during young adulthood.

**Fig. 1.**
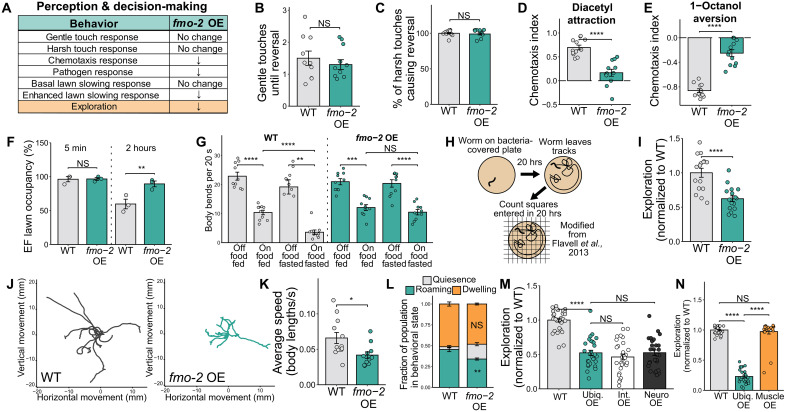
*fmo-2* overexpression alters *C. elegans* sensory perception and foraging behavior. (**A**) Summary of behavioral changes in *fmo-2* OE worms. (**B** and **C**) Quantification of the gentle (B) and harsh touch response (C). *N* = 10 worms per condition. (**D** and **E**) Quantification of the chemotaxis response to the attractant diacetyl (D) and the repellent 1-octanol (E). *N* ≥ 237 worms per condition. (**F**) Quantification of *E. faecalis* (EF) pathogen fleeing after 5 min and 2 hours of exposure. *N* ≥ 85 worms per condition. (**G**) Quantification of the basal and enhanced slowing response. *N* = 10 worms per condition. (**H**) Diagram of the exploration assay and (**I**) quantification of exploration. *N* ≥ 16 worms per condition. hrs, hours. (**J**) Traces of worm paths traveled over a 30-min video normalized to begin at point (0,0). *N* ≥ 10 worms per condition. (**K**) Average speed of worms in (J). (**L**) Quantification of worms in quiescent, roaming, and dwelling behavioral states. Significance compares each OE behavioral state to the WT control group. *N* ≥ 52 worms per condition. (**M**) Exploration of ubiquitous (*eft-3p::fmo-2*), intestinal (*elt-2p::fmo-2*), and neuronal (*rab-3p::fmo-2*) *fmo-2* overexpressors. *N* ≥ 25 worms per condition. (**N**) Exploration of ubiquitous (*eft-3p::fmo-2*), and muscle (*unc-54p::fmo-2*) *fmo-2* overexpressors. *N* ≥ 24 worms per condition. Bar plots show the mean and SEM. NS (not significant), *P >* 0.05; *P < 0.05, **P < 0.01, ***P < 0.001, and ****P < 0.0001. Significance for all panels except (F) and (L) is from a one-way ANOVA and Tukey post hoc test (unpaired, two-tailed). Panels (F) and (L) show a two-way ANOVA [fraction ~ genotype * behavioral state (L) and occupancy ~ genotype * time point (F)]. Most panels show one representative replicate, except that (D), (E), (F), and (L) plot all replicates together. Statistics for these panels include a Bonferroni correction for multiple comparisons. Raw data from three replicates can be found in Supplementary Data.

Having established no obvious differences in locomotor ability, we moved to a second panel of behaviors designed to assess mental state, sensory perception, and foraging decisions. Here, we observed multiple effects of *fmo-2* overexpression on behavior (Fig. 1A). With respect to sensory perception, no changes were observed in responses to gentle touch (Fig. 1B) or harsh touch (Fig. 1C), indicating normal mechanosensation. In addition to mechanosensation, *C. elegans* also exhibit behavioral responses to various chemicals in their environment (*48*). For example, WT worms will move toward volatiles like diacetyl, benzaldehyde, and isoamyl alcohol as well as avoid other chemicals, such as 1-octanol or 2-nonanone (*49*, *50*). Notably, the *fmo-2* OE exhibited greatly reduced chemotaxis responses to both an attractive compound (diacetyl; Fig. 1D) and to an aversive compound (1-octanol; Fig. 1E). This decreased sensitivity to chemical attractants and repellants could indicate either a deficit in the worms’ ability to detect these compounds or a general apathy toward these chemicals. More broadly, these results suggest a role for an intestinal metabolic enzyme in modifying behaviors governed by multiple neural circuits.

In addition to altered chemotaxis responses, we observed substantial changes to foraging and decision-making behaviors. To measure a type of negative environment decision-making behavior, we performed a pathogen-fleeing assay, where WT worms generally avoid a lawn of harmful bacteria (*51*). In congruence with the hypothesis that *fmo-2* OE worms are apathetic to negative environments, they showed higher occupancy than WT worms on a lawn of pathogenic *Enterococcus faecalis* bacteria after 2 hours of exposure (Fig. 1F; representative images in fig. S2A). To examine foraging behavior, we assessed both typical foraging behavior and the starvation response in *C. elegans* by measuring the movement speed of the worm upon finding food (*52*). The basal slowing response, which indicates the difference in movement speed of well-fed animals before and after they encounter a bacterial lawn, was unchanged between WT and OE animals, suggesting that they recognize food appropriately. However, the OE animals did not display an enhanced slowing response, which measures lawn-slowing in animals that have been fasted for 30 min before testing (Fig. 1G). This suggests that the OE animals are insensitive to the behavioral effects of brief fasting, potentially because *fmo-2* overexpression is already mimicking a chronic DR state. Together, these results indicate that changes to intestinal *fmo-2* activity are sufficient to modify an organism’s perception of and/or response to positive and negative environmental signals.

Last, we tested WT and OE worms on an assay of exploratory behavior (*53*). In *C. elegans*, exploratory behavior reflects the balance of the animal’s foraging decisions to explore or exploit their environment. Increased exploration can indicate dissatisfaction with the local environment and an increased drive to find better resources. Decreased exploration, on the other hand, can indicate satisfaction with the local environment and/or a decision to conserve resources (*53*–*56*). Oftentimes, animals facing an environmental stressor like nutrient scarcity (*57*), hypoxia (*58*), or temperature stress (*59*) will initially exhibit an increase in movement to escape the stressor. However, chronic exposure to these types of stressors, which is often required to extend life span, can decrease exploration in favor of conserving energy and resources (*54*, *56*, *60*, *61*). The assay to measure exploration in nematodes encompasses movement over time as represented by the tracks left by a single worm on a 35-mm-diameter plate completely covered with bacterial food. The worm is allowed to explore this novel environment, and after a set amount of time, the worm is removed and the plate with its tracks is overlaid with a grid of squares. The number of squares the worm entered is counted to quantify the region of the plate explored by the worm (Fig. 1H). In this exploration assay, we observed a 47% decrease in exploration in the OE relative to WT (Fig. 1I).

To validate this low-exploration phenotype using another method, we next recorded worm movement on 35-mm plates completely covered in a bacterial lawn and quantified mean velocity over 30 min. We observed that the paths taken by the *fmo-2* OE as well as their mean velocity correlated with the changes observed in the initial exploration assay: *fmo-2* OE worms stayed closer to their point of origin than WT worms and displayed lower mean velocity over the 30-min period (Fig. 1, J and K). This result, coupled with the behavioral results discussed above, suggests that *fmo-2* overexpressors exhibit substantial changes in response to environmental cues despite a normal ability to move and an increased life span.

To identify the mechanism of these behavioral changes, we chose to primarily focus on exploration for three reasons: (i) there is a robust change in exploration in the *fmo-2* OE compared to WT; (ii) this behavior reflects both sensory perception of the environment and decision-making in response to these cues; and (iii) exploration measures a behavior that can be adaptive in the face of acute and chronic stress-response pathways activated by longevity interventions. To begin interrogating how *fmo-2* expression modifies exploration, we first examined the amount of time a worm spends in three distinct behavioral states: quiescence, roaming, and dwelling (*54*). Quiescence has been equated to a sleeplike state, where worms do not move at all. Dwelling worms exhibit a local-search foraging pattern, where they turn frequently and stay in one area. Roaming worms turn infrequently and explore more widely from their point of origin. We observed decreased roaming and increased quiescence in the OE but saw no changes in dwelling behavior (Fig. 1L). These results indicate that *fmo-2* OE changes exploration by shifting behavioral state rather than globally decreasing movement, again pointing to a more nuanced mechanism of action.

To further test the relationship between *fmo-2* expression and low exploration, we performed RNA interference (RNAi) knockdown of *fmo-2* on WT and OE animals (fig. S2B). *fmo-2* RNAi did partially rescue the low exploration of the OE, supporting the idea that high *fmo-2* expression is responsible for its low-exploration phenotype. However, we were surprised to find that knocking down *fmo-2* decreased exploration in WT worms, rather than having no effect or even increasing exploration, as one might expect from a knockout (KO) and overexpression model. Successful knockdown with *fmo-2* RNAi was validated with quantitative polymerase chain reaction (qPCR) (fig. S2C).

After finding that *fmo-2* overexpression modifies behavior in addition to extending life span, we next asked whether these phenotypes were driven by *fmo-2* overexpression in the same or in different tissues. Because *fmo-2* is primarily an intestinal enzyme but behavior is primarily governed by the nervous system, we measured the exploratory behavior of neuronal-specific and intestinal-specific *fmo-2* overexpressors. We found that overexpression in either tissue was sufficient to decrease exploratory behavior (Fig. 1M). When we overexpressed *fmo-2* in the muscle, a tissue where *fmo-2* is not endogenously expressed, it had no effect on exploratory behavior (Fig. 1N). To ensure that overexpressing *fmo-2* in one tissue was not leading to compensatory effects on *fmo-2* expression in other tissues, we next crossed these tissue-specific *fmo-2* OE strains into an *fmo-2* KO background and again examined their exploratory behavior (fig. S2D). We found that, even in the *fmo-2* null background, overexpressing *fmo-2* in either the nervous system or the intestine led to a low-exploration phenotype comparable to the ubiquitous *fmo-2* overexpressor. However, much like RNAi-mediated knockdown of *fmo-2* (fig. S2B), we found the *fmo-2* KO control displayed greatly decreased exploration compared to WT animals. These results support a “goldilocks” model for *fmo-2* expression in behavior, where a limited window of expression results in normal exploration.

However, identifying that decreased *fmo-2* expression also decreases exploration makes the findings from the tissue-specific *fmo-2* overexpression strains (fig. S2D) difficult to interpret and led us to wonder about the tissue-specific effects of *fmo-2* KO on exploration. Reexpressing *fmo-2* under a neuronal or an intestinal specific promoter in the *fmo-2* null background could indicate the tissue-specific necessity of *fmo-2* for normal exploration (fig. S2D). However, this approach is limited in that rescuing *fmo-2* expression under a nonendogenous promoter leads to over- or underexpression of *fmo-2* in the rescued tissue, both of which attenuate exploration. To further test the tissue specificity of low *fmo-2* expression, we put neuronal-only and intestinal-only RNAi uptake strains on *fmo-2* RNAi. We observed that knocking down *fmo-2* in the intestine did decrease exploration (fig. S2E), suggesting that intestinal *fmo-2* is required for normal exploration. However, the neuronal-specific RNAi uptake strain had a low-exploration phenotype that was not additive with *fmo-2* RNAi, making the neuronal-specific knockdown results difficult to interpret (fig. S2E). Given that (i) changing *fmo-2* expression in either direction counterintuitively led to decreased exploratory behavior and (ii) understanding the effects of blocking a signal can provide insight into the effects of overexpressing that signal, we next decided to characterize in greater depth how knocking out *fmo-2* also modifies behavior.

**Fig. 2.**
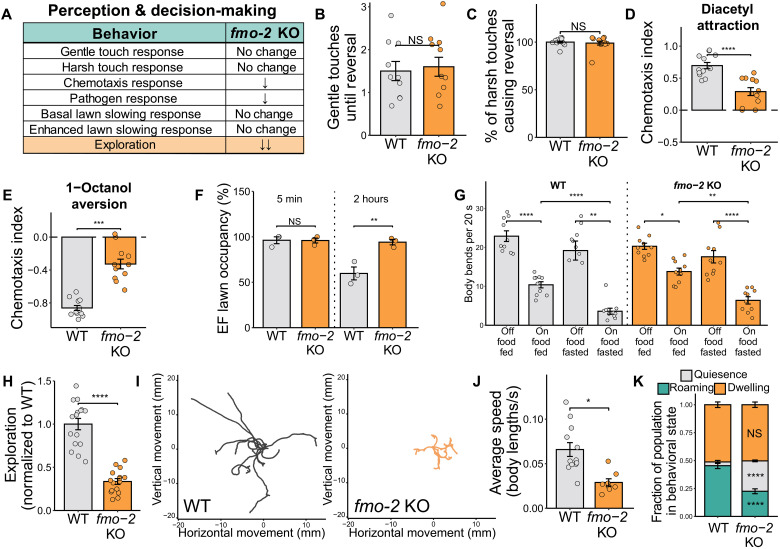
*fmo-2* knockout alters *C. elegans* sensory perception and foraging behavior. (**A**) Behavioral changes in *fmo-2* KO worms relative to WT controls. (**B** and **C**) Quantification of the gentle (B) and harsh touch response (C). *N* = 10 worms per condition. (**D** and **E**) Quantification of chemotaxis to the attractant diacetyl (D) and the repellent 1-octanol (E). *N* ≥ 237 worms per condition. (**F**) Quantification of *E. faecalis* pathogen fleeing after 5 min and 2 hours of exposure. *N* ≥ 85 worms per condition. (**G**) Quantification of the basal and enhanced slowing response. *N* = 10 worms per condition. (**H**) Quantification of exploration in WT and *fmo-2* KO worms. *N* ≥ 16 worms per condition. (**I**) Traces of worm paths traveled over a 30-min video normalized to begin at point (0,0). *N* ≥ 10 worms per condition. (**J**) Average speed of worms in (I). (**K**) Quantification of worms in quiescent, roaming, and dwelling behavioral states. Significance compares each OE behavioral state to the WT control group. *N* ≥ 52 worms per condition. Bar plots show the mean and SEM. NS, *P >* 0.05; *P < 0.05, **P < 0.01, ***P < 0.001, and ****P < 0.0001. Significance for all panels except that (G) and (F) is from a one-way ANOVA and Tukey post hoc test (unpaired, two-tailed). (K) and (F) show a two-way ANOVA [fraction of population ~ genotype * behavioral state (K) and lawn occupancy ~ genotype * time point (F)]. Panels (B), (C), (G), (H), (I), and (J) show one representative replicate. Raw data from all three replicates can be found in Supplementary Data. Panels (D), (F), and (K) plot data from three replicates together. Statistics for these panels include a Bonferroni correction for multiple comparisons.

To characterize the effects of *fmo-2* KO on behavior, we performed the same panels of assays we used to measure *fmo-2* OE movement capacity (fig. S3A) and behavior (Fig. 2A). We found that *fmo-2* KO did not alter movement capacity of *C. elegans* at day 1 of adulthood, as measured by maximum velocity, pumping, and thrashing assays (fig. S3, A to D). Much like the *fmo-2* OE, *fmo-2* KO animals also exhibited no differences in response to gentle or harsh touch (Fig. 2, B and C). However, the knockouts did display decreased chemotaxis responses to the attractant diacetyl and the repellant 1-octanol (Fig. 2, D and E). They also exhibited a blunted pathogen-fleeing response (Fig. 2F; representative images in fig. S3E). Unlike the *fmo-2* OE, the *fmo-2* KO behaved like WT worms in basal and enhanced lawn-slowing behavior (Fig. 2G). To validate the low-exploration phenotype of the *fmo-2* KO (Fig. 2H and fig. S2E), we again performed video assays and observed decreased exploration (Fig. 2I) and average speed (Fig. 2J). Consistent with the strong decreased exploration phenotype of the *fmo-2* KO, we also observed increased time spent in the quiescent behavioral state at the expense of time spent roaming (Fig. 2K). Together with our findings from the *fmo-2* OE, these results indicate that modifying *fmo-2* expression through either up-regulation or down-regulation results and similar blunted responses to various environmental cues. In addition, both *fmo-2* mutants exhibit changes in foraging patterns and behavioral state that can be recapitulated by modifying *fmo-2* expression in either the nervous system or the intestine. These findings are consistent with a mechanism in which *fmo-2* activity in either tissue changes metabolism in a way that modifies neural signaling and, in turn, alters behavior.

### *fmo-2* expression interacts with tryptophan metabolism to alter exploration

**Fig. 3.**
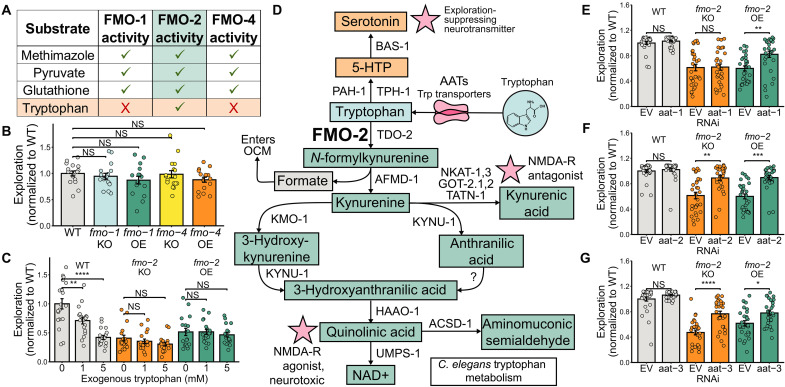
*fmo-2* expression interacts with tryptophan metabolism to alter exploratory behavior. (**A**) Summary of the enzymatic activities of *C. elegans* FMO-1, FMO-2, and FMO-4 on the substrates methimazole, pyruvate, glutathione, and tryptophan. *K*_cat_ and *K*_m_ values can be found in Supplementary Data. (**B**) Quantification of the exploration of *fmo-1* KO (*ok405*), *fmo-1* OE (*eft-3p::fmo-1*), *fmo-4* KO (*ok294*), and *fmo-4* OE (*eft-3p::fmo-4*) worms. *N* ≥ 16 worms per condition. (**C**) Exploration of WT, *fmo-2* KO, and *fmo-2* OE worms after supplementation from egg on plates with 0, 1, or 5 mM tryptophan in the agar. *N* ≥ 18 worms per condition. (**D**) Diagram of the tryptophan uptake and metabolism in *C. elegans*. NMDA-R, NMDA receptor. (**E** to **G**) Quantification of the exploration of WT, *fmo-2* KO, and *fmo-2* OE worms on EV or *aat-1* (E), *aat-2* (F), and *aat-3* (G) RNAi. *N* ≥ 29 (E), *N* ≥ 27 (F), and *N* ≥ 29 (G) worms per condition. In all bar plots, the top of the bar represents the mean of the population and error bars indicate the SEM. In all panels, NS, *P >* 0.05; *P < 0.05, **P < 0.01, ***P < 0.001, and ****P < 0.0001. Significance for all panels is from a one-way (B) or two-way [(C), (E), (F), and (G)] ANOVA and Tukey post hoc test (unpaired, two-tailed). All panels show one representative replicate. Raw data from all three replicates can be found in Supplementary Data.

Given that intestinal *fmo-2* over- and underexpression can modify both behavior and longevity (*62*), we next sought to establish the specificity of and substrate(s) responsible for these effects. To test for specificity, we asked whether modifying the expression of other FMOs with similar activities could also change exploration. We tested the behavior of *fmo-1* or *fmo-4* KO and OE worms because FMO-1 and FMO-4 are structurally the most similar to FMO-2. We found that *fmo-1* and *fmo-4* KO and OE worms exhibited normal exploratory behavior (Fig. 3B), suggesting that the behavioral role is specific to FMO-2. Upon testing the three enzymes for substrate specificity, we found that FMO-1 and FMO-4 act on many of the same substrates as FMO-2, with the notable exception of tryptophan, which is specific to *C. elegans* FMO-2 (Fig. 3A). These data are consistent with a model in which altered oxygenation of tryptophan, the only identified substrate unique to FMO-2, may be responsible for the behavioral effects of *fmo-2* expression.

Consequently, we sought to determine whether FMO-2’s activity on tryptophan or on any other individual substrate is involved in *fmo-2–*mediated behavioral change. To test whether the behavioral changes of *fmo-2* KO and OE are specific to tryptophan and or any other known FMO-2 substrates, we measured the effects of supplementing each established FMO-2 substrate on exploration. We found that only tryptophan supplementation dose dependently decreased WT exploration (Fig. 3C), with no significant effect from glutathione, methimazole, or pyruvate (fig. S4, A to C). Notably, the reduction in exploration observed in *fmo-2* KO and OE worms was not additive with the decrease caused by exogenous tryptophan, suggesting that altered tryptophan metabolism through *fmo-2* affects exploratory behavior via the same mechanism (Fig. 3C). Tryptophan supplementation did not affect thrashing or maximum velocity (fig. S4, E and F), indicating that these results are not due to diminished movement capacity. Together, these data indicate a specific role for the highly conserved amino acid tryptophan in *fmo-2–*mediated behavioral change.

To confirm that tryptophan uptake is required for exogenous tryptophan supplementation to decrease exploration, we grew WT worms on RNAi against the two best-validated *C. elegans* tryptophan transporters (Fig. 3D), *aat-2* and *aat-3* (*63*, *64*). We observed that worms with either transporter knocked down were resistant to the behavioral effects of exogenous tryptophan (fig. S4F), suggesting that tryptophan uptake into cells is required for decreased exploration. This indicates a metabolic mechanism of action, rather than behavioral change resulting from sensory perception of amino acid(s) in the environment. We next used qPCR to test whether changes in *fmo-2* expression indirectly modify tryptophan metabolism through compensatory changes in expression of other enzymes in the tryptophan metabolism pathway. We found that knocking out or overexpressing *fmo-2* had no significant effects on the expression of *tdo-2*, *afmd-1*, *kynu-1*, and *haao-1* relative to WT worms (fig. S4G). This suggests that any interactions between *fmo-2* and tryptophan metabolism are not a result of secondary transcriptional effects on other genes in this pathway.

Having established that tryptophan supplementation is sufficient to decrease exploration in WT worms, we next asked whether tryptophan is necessary for low exploration in the KO and OE worms. To reduce tryptophan uptake, we again used RNAi to knockdown amino acid transporters (AATs) with high similarity to mammalian homologs—*aat-1*, *aat-2*, and *aat-3* (*63*). We observed that knockdown of all three AATs tested partially rescued the exploratory behavior of the OE (Fig. 3, E to G), whereas *aat-2* and *aat-3* knockdown partially rescued the KO (Fig. 3, F and G). AAT-1 also transports the tryptophan metabolite kynurenic acid (*64*), suggesting a potential role for kynurenic acid in the decreased exploration of the *fmo-2* OE but not the KO. All RNAi knockdowns were validated with qPCR for expression of the targeted gene (fig. S4H). These data indicate that tryptophan uptake is at least partially required for *fmo-2* expression to modify exploration.

We next asked how *fmo-2* expression interacts with tryptophan metabolism to modify exploration. A previous work from our lab found that FMO-2 oxygenates tryptophan to form *N*-formylkynurenine in vitro (*24*). *N*-Formylkynurenine is then converted into kynurenine by formidase (*afmd-1*), which also releases formate as a by-product (Fig. 3D). This formate can enter the folate cycle, potentially altering the flux of the one-carbon metabolism (OCM) cycle and modifying life span (*24*). To test whether *fmo-2* modifies behavior indirectly through this interaction with OCM, we used RNAi to knock down *sams-1* and decrease S-adenosyl methionine synthesis. We observed no change in exploration after *sams-1* knockdown (fig. S4, I and J), indicating that the behavioral effects of *fmo-2* are more likely directly related to tryptophan metabolism rather than OCM. Again, these results suggest that (i) tryptophan metabolites likely play a key role in *fmo-2–*mediated behavioral change and (ii) the mechanisms of *fmo-2–*mediated longevity and behavioral change may be separable.

### Knocking out *fmo-2* suppresses exploration by modifying serotonin signaling

**Fig. 4.**
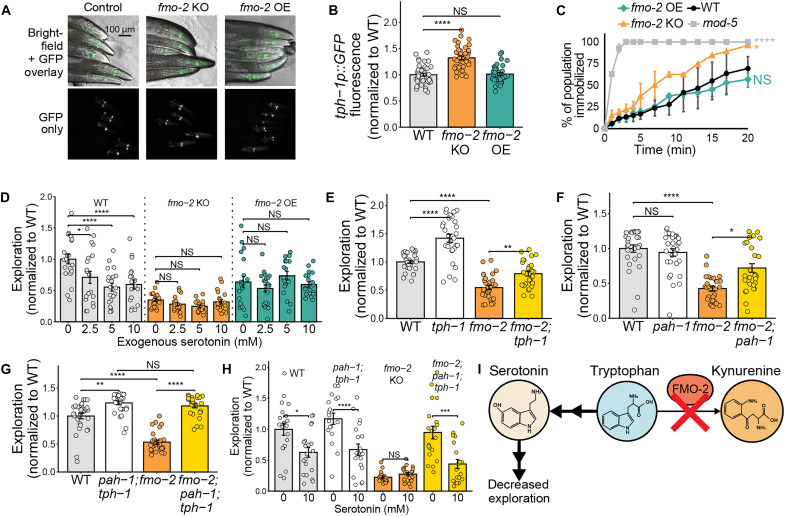
Knocking out *fmo-2* suppresses exploratory behavior by modifying serotonin signaling. (**A** and **B**) Representative images (A) and quantification of the mean head fluorescence (B) of *zdls13* (*tph-1p::GFP*), *fmo-2*(*ok2147*)*; zlds13*(*tph-1p::GFP*), and *fmo-2* overexpressor (*eft-3p::fmo-2*)*; zdls13* (*tph-1p::GFP*)*. N* ≥ 23 worms per condition. (**C**) Quantification of the immobilization of WT, *fmo-2* KO, *fmo-2* overexpressor, and *mod-5*(*n3314*) in 30 mM serotonin in M9 buffer over a 20-min period. *N* ≥ 128 worms per genotype. (**D**) Exploration of WT, *fmo-2* KO, and *fmo-2* OE worms on 0, 2.5, 5, and 10 mM exogenous serotonin from egg. *N* ≥ 18 worms per condition. (**E**) Exploration of WT, *tph-1*(*mg280*), *fmo-2*(*ok2147*), and *tph-1*(*mg280*)*; fmo-2*(*ok2147*) worms. *N* ≥ 28 worms per condition. (**F**) Exploration of WT, *pah-1*(*syb3601*), *fmo-2*(*ok2147*), and *pah-1*(*syb3601*); *fmo-2*(*ok2147*) worms. *N* ≥ 28 worms per condition. (**G**) Exploration of WT, *tph-1*(*mg280*)*; pah-1*(*syb3601*), *fmo-2*(*ok2147*), and *tph-1*(*mg280*)*; pah-1*(*syb3601*); *fmo-2*(*ok2147*) worms. *N* ≥ 27 worms per condition. (**H**) Exploration of WT, *tph-1*(*mg280*)*; pah-1*(*syb3601*), *fmo-2*(*ok2147*), and *tph-1*(*mg280*)*; pah-1*(*syb3601*); *fmo-2*(*ok2147*) worms on 0 or 10 mM exogenous serotonin from egg. *N* ≥ 19 worms per condition. (**I**) Working model of the mechanism through which *fmo-2* KO decreases exploration. In all bar plots, the top of the bar represents the mean of the population and error bars indicate the SEM. In all panels, NS, *P* > 0.05; *P < 0.05, **P < 0.01, ***P < 0.001, and ****P < 0.0001. Significance for all panels is from a one-way [(B), (C), (E), (F), and (G)] or two-way [(D) and (H)] ANOVA and Tukey post hoc test (unpaired, two-tailed). All panels show one representative replicate. Raw data from all three replicates can be found in Supplementary Data.

Having ruled out changes in OCM as a mechanism of *fmo-2–*mediated behavioral change, we asked whether the accumulation or depletion of any tryptophan-derived metabolites could lead to low exploration in these strains. On the basis of the role of *fmo-2* in the tryptophan metabolism pathway (Fig. 2D), we hypothesized that knocking out *fmo-2* may increase tryptophan flux toward serotonin, which is known to decrease exploration (*53*). To test whether *fmo-2* expression modifies serotonin production, we crossed the KO and OE with a transcriptional reporter for *tph-1*, the rate-limiting enzyme in neuronal serotonin synthesis from tryptophan (*65*). We observed a 40% increase in *tph-1* expression in the KO and no change in *tph-1* expression in the OE (Fig. 4, A and B). *tph-1* is expressed in two pairs of neurons in the head (ADF and NSM), and one pair of neurons near the vulva (HSN) that contribute to egg-laying (*65*). When the fluorescence intensity of *tph-1* was measured in individual serotonergic neurons, we again did not observe any changes in the OE (fig. S5, A to C). However, the KO displayed increased *tph-1* expression in the NSM but not in the ADF or HSN neurons (fig. S5, D and F). The NSM acts as a key signal in multiple foraging-related behavioral circuits (*52*, *55*, *66*). Serotonin production in the NSM, but not the ADF and HSN, has also been shown to increase quiescent behavior at the expense of roaming behavior (*67*), as is observed in the *fmo-2* KO worms (Fig. 2K). These data are consistent with higher serotonin production in the KO and no change in serotonin production in the OE.

Because increased transcription of *tph-1* does not necessarily lead to higher levels of serotonin, we further examined whether the behavioral changes mediated by *fmo-2* are linked to altered serotonin. To test the likelihood of high serotonin in the KO, we performed a serotonin immobilization assay in which worms are placed in a high concentration (30 mM) serotonin solution. Worms with high baseline levels of extrasynaptic serotonin, like the serotonin reuptake transporter KO *mod-5*, are immobilized faster than WT in this assay (*68*). We observed that the KO was immobilized faster than WT, whereas immobilization of the OE did not differ from WT (Fig. 4C). These results suggest the KO has elevated extrasynaptic serotonin availability whereas the OE does not. Consequently, the low exploration of the OE cannot be explained by altered serotonin availability, but the low exploration of the KO could be due to increased serotonin signaling.

To confirm whether increased serotonin availability is sufficient to suppress exploration and that the low exploration of the OE is not driven by flux of tryptophan away from serotonin, we supplemented WT, KO, and OE worms with exogenous serotonin. Exogenous serotonin decreased WT exploration but did not alter the exploration of the KO and OE (Fig. 4D). Serotonin supplementation did not decrease performance of WT worms in thrashing and maximum velocity assays, suggesting that the effects of serotonin are not a result of decreased movement capacity (fig. S4, G and H). These data suggest that high serotonin is not additive with low *fmo-2* in decreasing exploration. In addition, the inability of serotonin supplementation to rescue the OE indicates that low serotonin is not likely causing the OE’s behavioral change.

After finding that supplementing WT worms with exogenous serotonin is sufficient to phenocopy the low exploration of the KO, we next sought to test the necessity of serotonin signaling in the KO’s low-exploration phenotype. To test the necessity of serotonin, we first used RNAi to knockdown *tph-1* or *pah-1* expression in WT, KO, and OE animals. Like mammals, *C. elegans* produce serotonin in neurons and in peripheral tissues such as the intestine. Neuronal serotonin production requires *tph-1*, whereas peripheral serotonin production requires *pah-1* (*69*). Blocking *tph-1* partially rescued the exploratory behavior of the KO, and we observed an insignificant trend toward increased exploration in WT and OE worms on *tph-1* RNAi (*P* = 0.16 and *P* = 0.09, respectively) (fig. S5I). *pah-1* knockdown not only had no effect on exploration in the WT or OE animals but also partially rescued the exploration of the KO (fig. S5J). Both RNAi knockdowns were qPCR validated (fig. S5K). Together, these data indicate that decreasing serotonin synthesis in either the nervous system or in peripheral tissues partially reverses the effects of *fmo-2* KO on exploration. This again supports a model in which global metabolic changes driven by *fmo-2* expression modify neurotransmitter production to alter behavior.

To confirm our RNAi knockdown data, we knocked out either *tph-1*, *pah-1*, or both in the *fmo-2* KO and *fmo-2* OE background. Again, we observed that knocking out each gene partially rescued the exploration of the *fmo-2* KO (Fig. 4, E and F). However, when we knocked out both *tph-1* and *pah-1* in the *fmo-2* KO background, we observed a complete rescue of exploratory behavior to the level of the *pah-1; tph-1* control (Fig. 4G). Moreover, the exploration of *fmo-2; pah-1; tph-1* was reversed back toward baseline *fmo-2* KO exploration levels by supplementation with exogenous serotonin (Fig. 4H), suggesting that *pah-1; tph-1* knockout directly mediates KO exploration via serotonin availability.

To test whether serotonin availability is measurably higher in *fmo-2* KO worms compared to WT controls, we performed targeted metabolomics to measure serotonin levels in WT, *fmo-2* KO, *pah-1; tph-1*, *pah-1; tph-1; fmo-2*, and *mod-5* animals (fig. S5L). As previously reported, *pah-1; tph-1* animals had significantly lower levels of serotonin than WT (*69*). *fmo-2* KO animals displayed a nonsignificant trend toward higher serotonin abundance, and knocking out *pah-1* and *tph-1* in the *fmo-2* KO background restored serotonin in the *fmo-2* KO to the low level of the *pah-1; tph-1* controls. The serotonin reuptake transporter, *mod-5*, was included as a positive control, with the expectation that preventing serotonin reuptake may lead to a detectable increase in serotonin abundance. However, similarly to the *fmo-2* KO worms, we observed a nonsignificant trend toward increased serotonin in the *mod-5* KO strain. These results are consistent with a model where *fmo-2* KO and/or *mod-5* KO may increase local serotonin availability within the *C. elegans* nervous system, but local changes in a low-abundance metabolite are difficult to detect in whole-body samples.

Knocking out *pah-1*, *tph-1*, or *pah-1* and *tph-1* in *fmo-2* OE worms also increased exploration (fig. S5, M to O). However, the *fmo-2* OE’s exploration was not rescued to the level of the *tph-1* or *tph-1; pah-1* controls, and we did not observe a significant interaction between *fmo-2* overexpression and serotonin production on exploration relative to WT controls [two-way analysis of variance (ANOVA), *fmo-2* expression*serotonin synthesis]. We also tested the effect of blocking serotonin synthesis in another tryptophan/kynurenine metabolism mutant, *haao-1*. We found that knocking out *tph-1* and *pah-1* in the *haao-1* KO background increased exploration to the level of the *tph-1; pah-1* control (fig. S5P). This is consistent with our data that, although blocking serotonin synthesis can increase exploration in a variety of genetic backgrounds (WT and *fmo-2* OE), serotonin production is completely required for the low exploration of the *fmo-2* KO. Knocking out *haao-1* also did not interact with the effects of serotonin supplementation on exploration, suggesting that there is no strong interaction between serotonin signaling and kynurenine metabolism on exploration (fig. S5Q).

After finding that serotonin synthesis is required for *fmo-2* KO to decrease exploration and that the *fmo-2* KO displays increased *tph-1* transcription in the NSM neurons, we examined interaction between *fmo-2* knockdown and neuron-specific serotonin production in exploration. To test this, we measured the exploratory behavior of worms with *tph-1* knockout out in either the NSM neurons or the ADF neurons on empty vector (EV) or *fmo-2* RNAi (fig. S5R). We found that knocking out serotonin synthesis in either neuron type increased exploration relative to WT worms. *fmo-2* knockdown decreased exploration in WT and the ADF *tph-1* KO strains but not in the NSM *tph-1* KO strain. In concert with our *tph-1* transcriptional reporter data (fig. S5E), this suggests that NSM serotonin production is required for decreased *fmo-2* expression to suppress exploration. Together, these data support a model in which knocking out *fmo-2* modifies serotonin signaling in neurons and/or in the intestine, which consequently lowers exploration (Fig. 4I). This model indicates a role for Fmos in modulating a highly conserved neurotransmitter with broad impacts on health and behavior.

### Quinolinic acid signaling lowers exploration in the *fmo-2* overexpressor

**Fig. 5.**
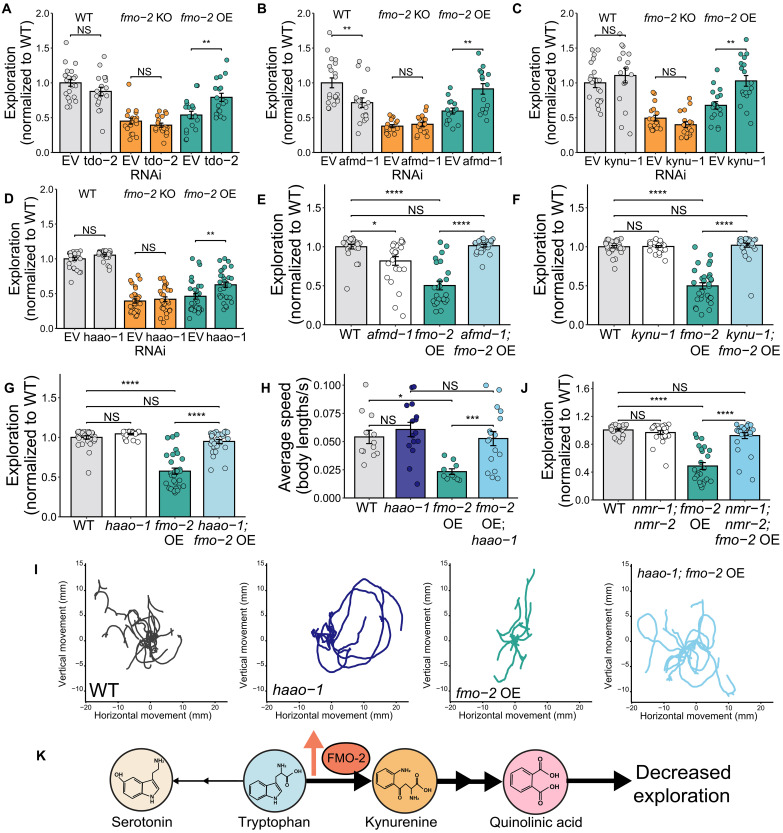
The kynurenine metabolites quinolinic and kynurenic acid contribute to exploratory behavior changes in the *fmo-2* overexpressor. (**A** to **D**) Exploration of WT, *fmo-2* KO, and *fmo-2* OE worms on EV or (A) *tdo-2*, (B), *afmd-1*, (C), *kynu-1*, and (D) *haao-1* RNAi. *N* ≥ 18 (A), *N* ≥ 16 (B), *N* ≥ 16 (C), and *N* ≥ 29 (D) worms per condition. (**E**) Exploration of WT, *afmd-1*(*tm4547*), *fmo-2* overexpressor, and *afmd-1*(*tm4547*)*; fmo-2* overexpressor worms. *N* ≥ 24 worms per condition. (**F**) Exploratory behavior of WT, *kynu-1*(*tm4924*), *fmo-2* overexpressor, and *kynu-1*(*tm4924*)*; fmo-2* overexpressor worms. *N* ≥ 30 worms per condition. (**G**) Exploration of WT, *haao-1*(*tm4627*), *fmo-2* overexpressor, and *haao-1*(*tm4627*)*; fmo-2* overexpressor worms. *N* ≥ 28 worms per condition. (**H** and **I**) Average speed (H) and paths traveled (I) over a 30-min video recording. *N* ≥ 10 worms per condition, and each path from a single worm was normalized to begin at point (0,0). (**J**) Exploration of WT, *nmr-1*(*ak4*)*; nmr-2*(*ok3324*), *fmo-2* overexpressor, and *nmr-1*(*ak4*)*; nmr-2*(*ok3324*)*; fmo-2* overexpressor worms. *N* ≥ 24 worms per condition. (**K**) Working model of how *fmo-2* overexpression decreases exploratory behavior. In all bar plots, the top of the bar represents the mean of the population and error bars indicate the SEM. In all panels, NS, *P* > 0.05; *P < 0.05, **P < 0.01, ***P < 0.001, and ****P < 0.0001. Significance in (A) to (H) and (J) is from a one-way [(E) to (H) and (J)] or two-way [(A) to (D)] ANOVA and Tukey post hoc test (unpaired, two-tailed). All panels show one representative replicate. Raw data from all three replicates can be found in Supplementary Data.

We next sought to interrogate the mechanism of tryptophan-mediated behavioral change in the *fmo-2* OE. Because the OE did not display decreased *tph-1* expression and was not rescued by exogenous serotonin, we wondered whether *fmo-2* OE could instead increase flux toward kynurenine-derived metabolites that decrease exploration. To test this hypothesis, we used RNAi to knockdown all genes involved in tryptophan-kynurenine metabolism. WT, KO, and OE worms were grown on either EV or *tdo-2*, *afmd-1*, *nkat-1*, *nkat-3*, *tatn-1*, *got-2.1*, *got-2.2*, *kmo-1*, *kynu-1*, *haao-1*, *acsd-1*, or *umps-1* RNAi for two generations, and then their exploratory behavior was measured. Successful knockdown of each gene was confirmed via qPCR (fig. S6A). The first hit from this RNAi screen was *tdo-2*, a gene that acts in the same step as *fmo-2* in *C. elegans* tryptophan metabolism (Fig. 3D). *tdo-2* knockdown did not alter WT or KO exploration but did partially rescue the OE (Fig. 5A). This finding is consistent with a model in which increased TDO-2 and/or FMO-2 activity pushes tryptophan toward the kynurenine pathway, which decreases exploration. Consequently, knocking down *tdo-2* partially compensates for *fmo-2* overexpression to rescue exploratory behavior.

Because knockdown of *afmd-1*, *kynu-1*, and *haao-1* only partially rescued the behavior of the OE, we validated our results using genetic knockouts. We found that knocking out *afmd-1* slightly decreased WT exploration but rescued *fmo-2* OE exploration to the level of *afmd-1* alone (Fig. 5E). Knocking out either *kynu-1* or *haao-1* fully rescued the *fmo-2* OE without affecting WT exploration (Fig. 5, F and G). *haao-1; fmo-2* OE worms also displayed similar movement patterns and average velocity to WT worms over a 30-min video recording (Fig. 5, H and I). The ability of these kynurenine metabolism enzyme knockouts to completely rescue the *fmo-2* OE indicates that the kynurenine-derived neuromodulator quinolinic acid is required for this strain’s low exploration.

We also used genetic knockouts to confirm our negative data from *umps-1* and *nkat-1* RNAi. The *fmo-2* OE was crossed into an *umps-1* KO background because NAD (nicotinamide adenine dinucleotide) plays a role in energy balance and longevity pathways (*70*, *71*), and we wanted to test whether altered energy availability might drive changes in exploration. However, crossing the OE into an *umps-1* KO strain also failed to rescue exploration (fig. S6J). Loss of *umps-1* in WT animals decreased exploration, further supporting a model where quinolinic acid production suppresses exploration. Much like with the *fmo-2* KO, the role of *fmo-2* OE in regulating neuromodulator availability implicates metabolically active tissues as important drivers of neural signaling and behavioral state.

The *haao-1* and *umps-1* KO data indicate that quinolinic acid is necessary for the low exploration of the OE and sufficient to decrease exploration in WT. However, we were surprised to see that blocking kynurenic acid production through knockdown of *nkat-1*, *nkat-3*, *tatn-1*, *got-2.1*, or *got-2.2* had no effect on exploratory behavior, considering that kynurenic acid plays a role in *C. elegans* feeding behavior (*72*) (fig. S6, B to F). To confirm this result, we also crossed the *fmo-2* OE into an *nkat-1* null background. *nkat-1* was chosen over the other kynurenic acid synthesis genes because *nkat-1* is expressed in the nervous system and is known to modify other behaviors in *C. elegans* (*64*, *73*). We observed a bimodal distribution in the exploration of the *fmo-2* OE; *nkat-1* worms. Approximately half of these animals were not affected by *nkat-1* knockout, whereas the other half of the population was rescued by blocking neuronal kynurenic acid synthesis (fig. S6K). This result could suggest that the buildup of kynurenic acid is responsible for the decreased exploration in some but not all OE worms. However, it is also possible that knocking down two or more of these genes simultaneously would be required to prevent kynurenic acid buildup and rescue exploration in all OE animals.

After finding that blocking quinolinic acid or kynurenic acid synthesis can rescue *fmo-2* OE exploration, we next asked whether supplementation of these metabolites or their precursors is sufficient to decrease exploration in WT animals. We found that exogenous quinolinic acid was sufficient to decrease exploration in WT worms but did not further decrease the exploration of *fmo-2* KO or OE (fig. S6L). Exogenous kynurenic acid led to an insignificant trend toward decreased exploration in WT at 5 mM and had no effect on the *fmo-2* KO or OE (fig. S6M). To test for interactions between kynurenine metabolism and serotonin signaling on exploratory behavior, we also supplemented *pah-1; tph-1* (− serotonin) animals with quinolinic acid. We found that quinolinic acid still dose-dependently decreased exploration in these worms, suggesting minimal interactions between serotonin availability and quinolinic acid on exploration (fig. S6N). To further explore how increased flux through kynurenine metabolism modifies exploration, we next supplemented WT worms with two other quinolinic acid precursors (kynurenine and 3-hydroxykynurenine). We found that both compounds dose-dependently decreased WT exploration (fig. S6, O and P). 3-Hydroxyanthranilate (fig. S6P) increased exploration at low concentrations (1 mM) but decreased exploration at higher concentrations (5 to 10 mM). Supplementation of these compounds did not decrease movement capacity in measurements of thrashing and maximum velocity, apart from 3-hydroxykynurenine, which decreased thrashing at 5 mM (fig. S6, Q to AA).

To further tease apart the relationship between quinolinic acid and kynurenic acid signaling in the exploration of the *fmo-2* OE, we next performed targeted metabolomics for kynurenic and quinolinic acid on WT, *fmo-2* OE, *haao-1*, *haao-1; fmo-2* OE, and *umps-1* animals (fig. S6, AB to AD). We found that, as expected, knocking out *haao-1* significantly decreased quinolinic acid abundance compared to WT. *fmo-2* overexpression did not change quinolinic acid abundance relative to WT, but the *haao-1; fmo-2* OE strain did display the low quinolinic acid levels of the *haao-1* control (fig. S6AB). However, we did not detect an increase in quinolinic acid in our *umps-1* control (fig. S6AC), suggesting that detecting increases in a low-abundance signal may not be feasible in whole-body tissue samples. When we measured kynurenic acid abundance, we observed increased levels in both the *haao-1* control and the *haao-1; fmo-2* OE strain but no significant difference in the *fmo-2* OE alone (fig. S6AD). Together, these metabolomics data could indicate that local changes in quinolinic acid signaling rather than global changes in quinolinic acid abundance may contribute to the low exploration of *fmo-2* OE. In addition, the increased abundance of kynurenic acid observed in the *haao-1; fmo-2* OE strain could indicate a complex interaction between kynurenic acid and quinolinic acid in this low-exploration phenotype.

Having established a role for quinolinic and kynurenic acid signaling in the low exploration of the *fmo-2* OE, we next examined the shared receptor of these two neuromodulators, glutamatergic *N*-methyl-d-aspartate (NMDA) receptors. Quinolinic acid agonizes NMDA receptors, which can lead to excitotoxicity of NMDA receptor–expressing neurons. In contrast, kynurenic acid is a competitive inhibitor at the glutamatergic binding site in NMDA receptors. *C. elegans* only have one NMDA receptor, a heterotetramer of the *nmr-1* and *nmr-2* subunits (*74*, *75*). Because both quinolinic acid synthesis and kynurenic acid synthesis affected exploration in *fmo-2* OE animals, it is imperative to better understand their roles. We overexpressed *fmo-2* in an *nmr-1; nmr-2* null background and measured exploratory behavior. We observed that knocking out the NMDA receptor completely rescues the exploration of *fmo-2* overexpressors (Fig. 5J), supporting a model in which local quinolinic acid signaling drives glutamatergic neuron excitotoxicity that decreases exploration in *fmo-2* OE (Fig. 5K).

### *fmo-2* expression effects exploration, other behaviors, and longevity through partially but not completely overlapping mechanisms

**Fig. 6.**
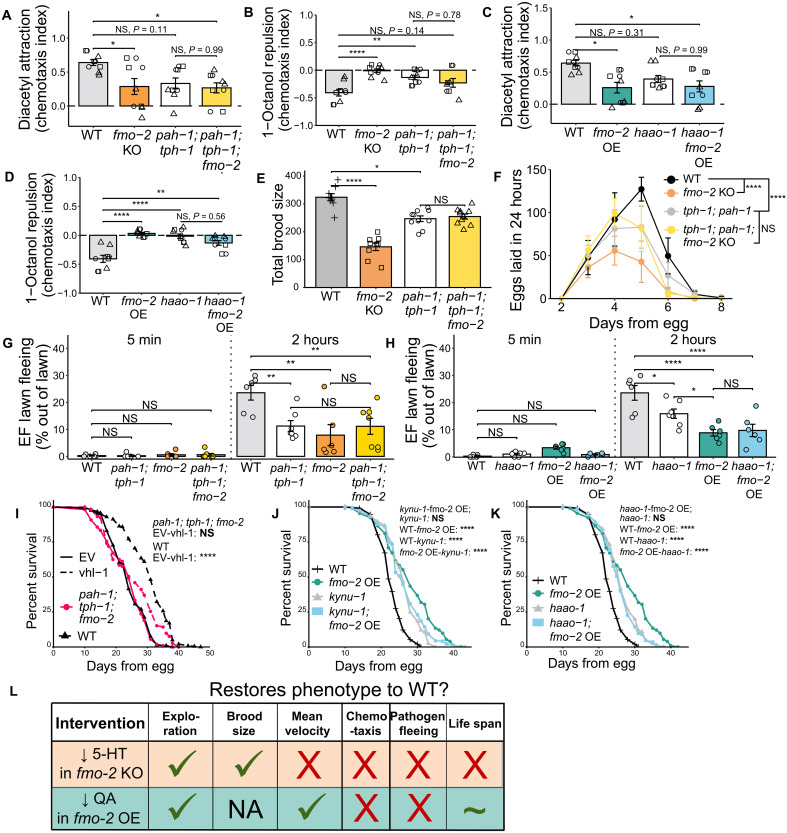
The mechanisms through which *fmo-2* expression affects exploratory behavior, other behaviors, and longevity are partially but not completely overlapping. (**A** to **D**) Chemotaxis to the attractant diacetyl [(A) and (C)] and repellent 1-octanol [(B) and (D)]. *N* ≥ 400 worms per condition. (**E** and **F**) Quantification of total brood size (E) and eggs laid each day of the reproductive life span (F) in WT, *pah-1*(*syb3601*); *tph-1*(*mg280*), *fmo-2*(*ok2147*), and *pah-1*(*syb3601*)*; tph-1*(*mg280*); *fmo-2*(*ok2147*) worms. *N* ≥ 7 worms per condition. (**G** and **H**) Quantification of *E. faecalis* pathogen fleeing after 5 min and 2 hours of exposure. *N* ≥ 350 worms per condition. (**I**) Survival curve of WT and *tph-1*(*mg280*)*; pah-1*(*syb3601*); *fmo-2*(*ok2147*) worms on EV or *vhl-1* RNAi. *N* ≥ 229 worms per condition. (**J**) Survival curve of WT, *kynu-1*(*tm4924*), *fmo-2* overexpressor, and *kynu-1*(*tm4924*)*; fmo-2* overexpressor worms. *N* ≥ 226 worms per condition. (**K**) Survival curve of WT, *haao-1*(*tm4627*), *fmo-2* overexpressor, and *haao-1*(*tm4627*)*; fmo-2* overexpressor worms. *N* ≥ 226 worms per condition. (**L**) Summary of the behavioral and aging-related phenotypes that were rescued by blocking serotonin (5-HT) synthesis in *fmo-2* KO worms or by blocking quinolinic acid (QA) synthesis in *fmo-2* OE animals. NA, not applicable. Bar plots show the mean and SEM. NS, *P* > 0.05; *P < 0.05, **P < 0.01, and ****P < 0.0001. Significance in (A) to (F) is from a one-way ANOVA and Tukey post hoc test (unpaired, two-tailed). Significance in (G) and (H) is from a two-way ANOVA (lawn occupancy ~ genotype * time point) and Tukey post hoc test (unpaired, two-tailed). Significance in (I) to (K) is from a log-rank test comparing median survival. Panels (E) and (F) show one representative replicate. Raw data from all three replicates can be found in Supplementary Data. Panels (A) to (D) and (G) to (K) plot data from three replicates together. Statistics for these panels include a Bonferroni correction for multiple comparisons.

After determining that blocking serotonin production rescues KO animal exploration and blocking quinolinic acid synthesis rescues OE animals, we wondered whether these manipulations could also rescue the other behavioral changes observed in these strains (Figs. 1 and 2). To answer this question, we first measured the chemotaxis responses of *fmo-2; pah-1; tph-1* and *haao-1; fmo-2* OE to attractive and repellant compounds. We found that blocking serotonin production decreased attraction to diacetyl (Fig. 6A) and reduced repulsion from 1-octanol (Fig. 6B) independently of *fmo-2*. All three strains showed a similarly reduced chemotaxis response to the attractant diacetyl (Fig. 6A). However, knocking out *pah-1* and *tph-1* in the *fmo-2* KO background did rescue the KO response to 1-octanol to the level of the *pah-1; tph-1* strain (Fig. 6B). We also measured the chemotaxis responses of *haao-1* and *haao-1; fmo-2* OE. *haao-1* exhibited a decreased response to both the attractant and the repellant to a degree similar to the *fmo-2* OE. Consequently, knocking out *haao-1* did not rescue the chemotaxis behavior of the OE (Fig. 6, C and D). These data suggest that modifying multiple points in the tryptophan metabolism pathway, through the activity of *fmo-2* or other key enzymes, is sufficient to modify behavioral outcomes.

To further test whether tryptophan metabolism is a common mechanism of *fmo-2–*mediated behavioral and physiological change, we looked at reproductive egg-laying behavior in the *fmo-2* KO and the *fmo-2; pah-1; tph-1* strains. A previous work on the role of *fmo-2* in *C. elegans* shows that knocking out *fmo-2* decreases brood size or reproductive capacity, whereas overexpressing *fmo-2* has no effect (*62*). Much like in the chemotaxis assay, *pah-1; tph-1* displayed decreased brood size relative to WT. Knocking out *tph-1* and *pah-1* in the *fmo-2* KO background rescued the brood size of the KO to the level of *pah-1; tph-1* (Fig. 6, E and F). These results indicate that the mechanism through which *fmo-2* KO alters exploration and reproduction may partially overlap. However, this was not the case for pathogen-fleeing behavior. As previously observed, both the KO and OE were less likely to flee a pathogenic lawn of *E. faecalis* than WT worms. Similarly to our chemotaxis response results, the modifications to tryptophan metabolism that rescued *fmo-2* KO and OE exploration (*pah-1; tph-1* knockout and *haao-1* knockout, respectively), resulted in decreased pathogen fleeing independently of *fmo-2* expression. Consequently, these interventions did not rescue the pathogen response behavior of the KO or OE (Fig. 6, G and H). Overall, these data suggest that the kynurenine metabolic pathway has multiple distinct mechanisms, including *fmo-2* OE, where modulation of this pathway affects behavior. However, altered serotonin signaling may contribute to both the reproduction and exploration phenotypes of the KO.

To test how behavior and longevity are linked, we asked whether the mechanism of *fmo-2*–mediated behavioral changes is distinct from *fmo-2–*mediated effects on longevity. *fmo-2* is required for hypoxia to extend life span in *C. elegans* (*62*). Because high serotonin drives behavioral changes in the *fmo-2* knockout, we asked whether high serotonin is also the reason that *fmo-2* KO is not long-lived under hypoxia. To answer this question, we examined the life span of *pah-1; tph-1*, and *fmo-2; pah-1; tph-1* on EV and *vhl-1* RNAi, a hypoxia mimetic. Successful RNAi knockdown was validated with qPCR (fig. S7A). We found that the life spans of both WT and *pah-1; tph-1* worms were extended by *vhl-1* RNAi (fig. S7B) but that *fmo-2* KO and *fmo-2; pah-1; tph-1* life spans were no longer extended by *vhl-1* RNAi (Fig. 6I and fig. S7C). This is consistent either with *fmo-2* acting downstream of serotonin signaling in hypoxia-mediated longevity or with *fmo-2* affecting hypoxia-mediated longevity through a serotonin-independent mechanism. This result also indicates that the effects of *fmo-2* KO on exploratory behavior and on longevity are separable.

Although *fmo-2* activity is required for environmental interventions like hypoxia to extend life span, overexpression of *fmo-2* is also sufficient to extend life span in *C. elegans* (*62*). To test whether the longevity phenotype of the *fmo-2* overexpressor is separable from the low-exploration phenotype, we measured the life span of *fmo-2* OE in the *kynu-1* KO or *haao-1* KO backgrounds. Notably, work from the Sutphin lab found that both *kynu-1* and *haao-1* knockouts are long-lived due to a buildup of the kynurenine-derived metabolite 3-hydroxyanthranilic acid (3HAA), which activates the oxidative stress response (*76*). We find that both *kynu-1* and *haao-1* extended life span relative to WT but were not as long-lived as the *fmo-2* OE (Fig. 6, I to K). Life spans of *kynu-1; fmo-2* OE and *haao-1; fmo-2* OE were not different from *kynu-1* or *haao-1* alone but shorter-lived than the *fmo-2* OE (Fig. 6, J and K). Consequently, rescuing the exploratory behavior of the *fmo-2* OE does mitigate the longevity benefits of *fmo-2* OE but does not suppress life span to the level of WT worms. Together, these findings highlight (i) the complex interplay between behavior and longevity and (ii) the essential role of tryptophan metabolism in both the behavioral and life-span effects of *fmo-2*.

Overall, these data indicate that maintaining regular flux through tryptophan metabolism—either through the activity of FMO-2 or other key enzymes—is essential for typical behavioral and aging-related outcomes. FMO-2 plays a critical role in this metabolic pathway as knocking out *fmo-2* modifies exploratory behavior and brood size requiring serotonin signaling. However, manipulations that restore some elements of WT-like behavior in the *fmo-2* KO, like blocking serotonin synthesis, disrupt the balance of tryptophan metabolism in other ways, leading to distinct behavioral effects (Fig. 6L). Similarly, the effects of *fmo-2* OE on exploratory behavior can be rescued by blocking quinolinic acid synthesis, but this intervention also modifies tryptophan metabolism in a manner that has consequences on chemotaxis and pathogen-fleeing behaviors (Fig. 6L). Last, many of these changes to tryptophan metabolism through *fmo-2* and other pathway components interact with longevity as well as multiple behaviors. Ideally, this circuit could be manipulated in a way where health span and life span are extended but WT-like behaviors are preserved. The closest intervention to meeting this goal may be the long-lived *haao-1; fmo-2* OE worms, which maintain some of the longevity benefits of *fmo-2* OE but display normal exploratory behavior. Together, these results not only provide a promising start in optimizing metabolic pathways for both health and behavioral goals but also highlight the additional work needed to untangle the effects of longevity interventions on behavioral and other physiological adaptations.

## DISCUSSION

In this work, we establish multiple behavioral changes that occur in response to modified expression of the longevity gene *fmo-2* in *C. elegans* (Figs. 1 and 2). Notably, we do not observe opposite behavioral effects of *fmo-2* KO and OE, as one might expect from a KO and overexpression model. Together, our data suggest a model in which altering *fmo-2* expression in either direction decreases exploratory behavior by changing flux through tryptophan metabolism (Fig. 3). In the case of the *fmo-2* KO, tryptophan that would normally be oxygenated into *N*-formylkynurenine is instead diverted toward increased serotonin synthesis. Elevated serotonin is sufficient to decrease exploration in WT worms and blocking serotonin production is sufficient to fully rescue the exploration of the KO (Fig. 4). In contrast, the OE alters flux of tryptophan toward kynurenine-derived metabolites including the NMDA receptor agonist quinolinic acid. Blocking any gene required for quinolinic acid synthesis and knocking out the NMDA receptor itself is sufficient to rescue exploratory behavior in *fmo-2* OE worms (Fig. 5). After identifying manipulations to the tryptophan metabolism pathway that can rescue the exploratory behavior of the KO and OE, we tested whether these manipulations also rescue other behavioral and longevity phenotypes of each strain. We found that knocking out and overexpressing *fmo-2* affects multiple behaviors and longevity through partially overlapping and partially distinct mechanisms (Fig. 6). Collectively, our data indicate a broad role for *C. elegans fmo-2* in multiple areas of physiology, including behavior, reproduction, and longevity.

Previous works on FMOs in *C. elegans* and in mammalian models have primarily focused on the role of FMOs in the immune response (*77*, *78*), endogenous metabolism (*26*, *28*, *79*, *80*), and, more recently, in health and longevity (*24*, *29*, *62*, *81*). However, *C. elegans fmo-2* was recently identified in a screen for genes required for aversion-resistant ethanol seeking behavior (*82*). Moreover, two environmental conditions that induce *fmo-2*, hypoxia (*83*) and DR (*84*), have also been found to modify behavioral responses in *C. elegans*. FMOs have also been proposed to play a role in neurodegeneration in mammals (*26*, *85*). In this work, we identify multiple behavioral responses that require *fmo-2*, suggesting a broad role for FMOs in modulating behavior.

Notably, the behavioral effects of both knocking out and overexpressing *fmo-2* interact with tryptophan metabolism. We were initially surprised to find that knocking out *fmo-2* could be sufficient to modify serotonin signaling, given that other enzymes such as *tdo-2* can also metabolize tryptophan into *N-*formylkynurenine. However, *C. elegans tdo-2* is primarily expressed in the hypodermis whereas *fmo-2* is lowly expressed in the nervous system and highly expressed in the intestine (*86*). This could suggest that tryptophan metabolism can have varied effects on behavior in different tissues. To better determine the tissue-specific effects of *fmo-2* on behavior, future work should examine how neuronal-specific or intestinal-specific *fmo-2* knockout affects behavior. In addition, only about 1 to 2% of tryptophan is thought to be metabolized into serotonin and other non-kynurenine metabolites (*87*), suggesting that a small change in tryptophan availability could lead to amplified changes in signaling from trace neuromodulators like serotonin. More work in this area is needed to understand both the tissue-specific effects of altered tryptophan metabolism and in understanding the movement of tryptophan-derived metabolites between tissues. In addition, future work should investigate any potential relationships between the regulation of and interactions between serotonin and kynurenine-derived neuromodulators on behavior. Last, although the work presented here explores how changes in *fmo-2* expression affect behavior through changes in tryptophan metabolism, the mechanistic details and neural circuits driving these behaviors remain unclear. More work is needed to uncover how tryptophan-derived metabolites are transported between tissues in these strains, and which serotonin-producing and quinolinic acid–producing tissues and cells are affected by modifications to *fmo-2* expression. This type of circuit-mapping work is of particular interest given that changes in quinolinic acid abundance were not detectable through global metabolite measurement. This could indicate that local alterations in signaling through this trace neuromodulator are key to its role in exploration.

A growing body of research has also linked dysregulated tryptophan metabolism and elevated quinolinic acid with depression, schizophrenia, and neurodegeneration (*87*–*90*). These investigations of the relationship between kynurenine metabolites and mental state often focus on the activity of indoleamine dioxygenases (IDOs) and tryptophan dioxygenases (TDOs) in this pathway. However, the finding that *fmo-2* overexpression can drive this type of dysregulated tryptophan metabolism suggests a potentially overlooked role for FMOs in modulating behavior. A previous work from our lab has found that tryptophan is also an in vitro substrate of mammalian Fmo5 (*24*), suggesting that the effects of *C. elegans* FMO-2 on tryptophan metabolism could be translatable to mammalian models. Much like *C. elegans fmo-2*, mammalian Fmo5 is lowly expressed in brain tissue (*91*) and highly expressed in the liver and intestine in mice and humans (*92*).

In addition to highlighting a role for FMOs in mental state, this work indicates the importance of considering pleiotropic effects of potential longevity interventions. Much like *fmo-2* overexpression, many other genetic and pharmaceutical longevity interventions target stress-response pathways that coordinate broad physiological changes across multiple tissues (*93*, *94*). Although it is relatively common to measure the effects of new longevity interventions on movement and reproductive capacity (*31*, *95*–*97*), other more nuanced changes in behavior are not often examined.

Although this work primarily focuses on exploratory behavior in the context of the longevity gene *fmo-2*, at least one other longevity intervention, XBP-1s overexpression, also decreases exploration in *C. elegans* (*98*). Synthesis of the neurotransmitter tyramine is required for *xbp-1* to modify exploration, suggesting that this behavioral output could be driven by multiple molecular mechanisms in different long-lived strains. However, it remains unclear whether low exploration consistently correlates with longevity as *haao-1* and *kynu-1* knockouts are also long-lived (*76*) but have no effect on exploration (Fig. 4). It is also possible that some longevity interventions mimic pathways activated under chronic stress and consequently lead to decreased exploration as a behavioral strategy to conserve resources. By contrast, other longevity interventions could activate components of the acute stress response, leading to increases in roaming and exploration observed during the acute behavioral response to many stressors (*57*–*59*, *61*). Future work should examine how additional longevity interventions affect exploration to determine whether this behavior correlates or decouples from life span. In addition, the effects promising longevity interventions on a greater variety of behavioral assays should be examined to determine which interventions are most specific to aging and whether additional concurrent interventions may be required to mitigate negative behavioral effects. Overall, additional work is needed to (i) better understand the role of FMOs in mental state and (ii) consider the behavioral effects of additional promising longevity interventions.

## MATERIALS AND METHODS

### Experimental design

Exact *N*s for each experiment are included in the source data files, and minimum *N*s for each plot are included in the figure legends. Sample sizes were determined using a power calculation and expected differences between control and experimental groups to achieve a 95% chance of detecting the expected change. In all experiments, condition numbers were randomized before quantification. When possible, the experimenter performing the experiment was also blinded to treatment differences between groups.

### Strains and growth conditions

*C. elegans* were cultivated using standard, previously described methods (*62*). Briefly, all worms were maintained at 20°C on solid nematode growth media (NGM). Worms were fed *Escherichia coli* OP50 throughout life, except in experiments using double-stranded RNAi (*E. coli* HT115). Worm transfers were conducted using a platinum wire unless otherwise specified. RNAi constructs used are listed in table S1, and strains used are listed in table S2. All genotypes were confirmed using PCR, and all RNAi imaging hits were sequence validated and qPCR validated before use.

### Pumping assays

Pumping was measured in day 2 gravid adults, as previously described (*62*). The number of pumps per 30 s was measured in at least 10 worms under 100x magnification. Because food availability is known to change pumping rate, only worms on the bacterial lawn were considered in this assay. Data were plotted by R version 4.3.1 and Adobe Illustrator 2022.

### Thrashing assays

Thrashing was measured in day 2 gravid adults, as previously described (*62*). Briefly, ~10 worms were placed in a droplet of M9 solution. Once worms began moving at a maximum rate, their body bends were counted for 30 s. Data were plotted by R version 4.3.1 and Adobe Illustrator 2022.

### Thirty-minute video recordings for velocity and movement patterns

On day 1 of adulthood, 10 to 15 worms of each genotype were placed in the center of a 35-mm petri dish that was completely covered in 350 μl of OP50 bacteria [bacteria were grown overnight to an optical density at 600 nm (OD_600_) of 3.0]. Worms were placed above the bright light source at the base of the microscope used for video recording for 10 min to acclimate to the light, and then 30-min video recordings were started. Videos were taken at 3.65x magnification and at 1 frame/s on a Leica M165F microscope using Leica Application Suite X (LASX) software and 4x4 binning. The videos were analyzed using the wrMTrck multiple object tracker plugin (wrMTrck_Batch v1.04) on FIJI (*99*) (FIJI Is Just ImageJ bundled with 64-bit Java 1.8.0) to measure average speed, maximum speed, worm area, total distance traveled, and distance from the point of origin. Raw *x* and *y* coordinates were also exported from wrMTrck to create the plots of worm paths over the course of the 30-min videos. Not all worm paths lasted the full 30-min duration of the video due to fleeing and collisions. In cases when worms fled the plate and were no longer visible during the video, the data from that worm were included if the on-plate duration was ≥10 min. In cases where worms collided and wrMTrck failed to correctly determine which worm was which after the event, the data for those worms before the collision were included if the duration was ≥10 min. In all data analysis using wrMTrck data, we confirmed that there was no significant difference in average path duration per condition. To generate path plots, the starting location of each worm was normalized to *x* = 0, *y* = 0. For each subsequent frame, the normalized *x*, *y* coordinate was determined by calculating the change in *x* and *y* position from the preceding to the current frame. Data were plotted by R version 4.3.1 and Adobe Illustrator 2022.

### Gentle touch response assay

Measuring the response of different strains to a gentle touch (eyebrow hair) was performed as previously described (*100*). Ten day 2 gravid adult worms were each stroked 10 times with an eyebrow hair, and touches were alternated from head to tail. Because no differences were observed between anterior and posterior touch response in any strain, head and tail touches were combined in calculating the number of touches until a response. A response was considered a change in movement direction away from the touched part of the worm or ceasing movement toward the direction of the touch. The average number of touches until a response was calculated for each worm. Data were plotted by R version 4.3.1 and Adobe Illustrator 2022.

### Harsh touch response assay

Measuring the response of different strains to a harsh touch (platinum wire pick) was performed as previously described (*100*). Ten day 2 gravid adult worms were each touched 10 times with a platinum wire pick, and touches were alternated from head to tail. Because no differences were observed between anterior and posterior touch response in any strain, head and tail touches were combined in calculating the percent of touches that elicited a response. A response was considered a change in movement direction away from the touched part of the worm or ceasing movement toward the direction of the touch. The percent of harsh touches that produced a response was calculated for each worm. Data were plotted by R version 4.3.1 and Adobe Illustrator 2022.

### Chemotaxis assay

For chemotaxis assays, unseeded NGM plates were separated into four quadrants using a marker on the bottom of the plate. A circle with a 5-mm radius was drawn in the center of the plate, and within each quadrant, a dot was marked in the middle of the quadrant 5 mm from the edge of the plate rim. Five microliters of 0.5 M sodium azide was pipetted onto each of the four dots (one per quadrant). Two quadrants opposite from one another were designated as control quadrants, and two were designated as treatment quadrants. In the treatment quadrants, 10 μl of diacetyl or 1-octanol was pipetted on top of the sodium azide. In the control quadrants, 10 μl of ethanol was pipetted on top of the sodium azide. Immediately after preparing the plates, gravid adults were washed off NGM plates in M9 and pelleted at 1000 rcf for 1 min. At least 40 worms per plate were then pipetted onto the 5-mm radius circle in the center of each chemotaxis plate. After 1 hour, the number of worms in the control and treatment quadrants was counted under a dissection microscope. Chemotaxis Index was calculated by subtracting the number of worms on all control quadrants from the number of worms on all treatment quadrants and then dividing that by the total number of worms on the plate. The attractant diacetyl and the repellent 1-octanol were applied at a dilution of 1:1000 compound to ethanol. Data were plotted by R version 4.3.1 and Adobe Illustrator 2022.

### Pathogen-fleeing assay

A flask containing 1x Brain Heart Infusion Broth (BHI, BD Difco) supplemented with rifampicin (100 μg/ml; Thermo Scientific Chemicals) was inoculated with *E. faecalis* OG1RF (American Type Culture Collection) and incubated for 18 hours with shaking at 37°C. From this culture, 100 μl of the 1x *E. faecalis* culture was seeded onto 35-mm BHI agar (BD Difco) plates containing rifampicin and a palmitic acid fence (0.5 g of palmitic acid in 50 ml of 100% ethanol), followed by incubation at 37°C for 18 hours. The palmitic acid fence was added to prevent worms from fleeing onto the sides of the plate and desiccating. After incubation, the plates were allowed to cool to room temperature before adding the worms. On day 1 of adulthood, ~100 worms were placed in the center of freshly prepared *E. faecalis* lawns and incubated at 25°C until imaging at 5-min, 2-hour, and 20-hour time points. Bright-field images were captured at each time point at 0.365x magnification using a Leica fluorescence microscope. The images were analyzed using ImageJ software, where the number of worms on and off the *E. faecalis* lawn was counted, and the percentage of lawn occupancy was calculated. Data were plotted by R version 4.3.1 and Adobe Illustrator 2022.

### Basal and enhanced slowing response assay

The basal and enhanced lawn slowing response assay was conducted as previously described by Sawin *et al.* (*52*). All measurements were performed on standard 60-mm diameter NGM plates, either seeded with 200 μl of *E. coli* OP50 in a ring on the outside of the plate or unseeded under control conditions. Plates were allowed to dry overnight before the assay. To measure the locomotory rate of fed animals, well-fed gravid worms on day 1 of adulthood were transferred off NGM plates and rinsed twice in M9 buffer. Approximately five worms at a time were then pipetted into the unseeded portion of the plate, and residual buffer was absorbed with a Kimwipe. After 5 min of acclimatization, the body bends of each animal were recorded over 20-s intervals until all animals had entered the region containing bacteria. To measure the behavior of fasted animals, the same procedure was followed but worms were first transferred to an NGM plate without food for 1/2 hour. Data were plotted by R version 4.3.1 and Adobe Illustrator 2022.

### Brood size assay

For each replicate, 10 worms per condition from a timed egg-lay were plated individually on 35-mm NGM plates as L4s, before egg-laying began. Once egg-laying began, mothers were transferred daily to new plates until no new eggs were laid for 2 days. Eggs were allowed to grow to L3 before counting. Worms that fled up the sides of the plate and could not be recovered during the assay were excluded from measurements of total brood size. However, preceding days in which the worm was on the plate for a full 24 hours were included in calculations of average eggs laid per day. Data were plotted by R version 4.3.1 and Adobe Illustrator 2022.

### Exploration assays

Exploration assays were performed as previously described by Flavell *et al.* (*53*). Individual worms from a timed egg-lay were transferred at young adulthood to a 35-mm NGM agar plate completely covered with *E. coli* OP50 (350 μl). Images were taken of each plate using an iPad attached to a bright-field microscope. A custom ImageJ java script was used to enhance the contrast of each image to an equal degree to improve visibility of worm tracks through the bacteria and to overlay a grid of uniform size across the image. The number of squares entered by the worm tracks was counted for each worm, with the maximum number of squares possible being 109. Twenty to 30 worms were assayed for each condition. Worms that fled the plate during the assay were excluded from analysis. Data were plotted by R version 4.3.1 and Adobe Illustrator 2022.

### Exploration assays with metabolite/compound supplementation

For all supplemented compounds, the specified dose of the compound was incorporated into the agar of 60-mm NGM plates, and worms were subjected to a timed egg-lay and grown on these specialty plates from egg. At young adulthood, worms were removed from the supplemented plates and placed on exploration plates (described above). The exploration assay was performed as indicated, with the exception that images were taken after 3 hours to reduce regression to control-like behavior after many hours off of plates with the supplemented compound. Data were plotted by R version 4.3.1 and Adobe Illustrator 2022.

### Behavioral state assays

On day 1 of adulthood, 10 to 20 worms under each condition were transferred to a standard 60-mm NGM plate seeded with *E. coli* OP50 bacteria. Animals were allowed to acclimate to the new plate for 30 min, and then an iPad attached to a bright-field microscope was used to record a 15-s video of the plate. An experimenter blinded to the plate condition then scored each worm on the plate as roaming (moving quickly with few turns), dwelling (moving around a local area with frequent turns), or quiescent (minimal to no movement) over the 15-s period. The percentage of the population in each state during that period was then calculated. Data were plotted by R version 4.3.1 and Adobe Illustrator 2022.

### Generating tissue-specific *fmo-2* overexpressors

Plasmid construction and microinjection were conducted by Suny Biotech to generate both tissue-specific *fmo-2* overexpression constructs. In designing the plasmids, we used the *elt-2* promoter to drive genomic DNA of FMO-2::SL2::GFP (green fluorescent protein) in the intestine. To express genomic DNA of FMO-2::SL2::GFP in neurons, we used the *rab-3* promoter. All plasmids were verified via restriction digest and Sanger sequencing, and ApE files are available upon request. Plasmids were microinjected by Suny Biotech using the coinjection marker myo-2p::GFP, and three transgenic lines were tested in each experiment.

### RNAi knockdown

For most RNAi knockdowns, worms were placed on the RNAi for two generations to ensure maximal knockdown. However, *sams-1* RNAi severely stunted development, so worms were placed on *sams-1* RNAi at the L3 stage then assayed as gravid adults. All RNAi clones were taken from the Vidal RNAi library. All RNAi was sequence verified, and successful knockdown was also qPCR validated.

### qPCR for RNAi validation

RNAi was isolated from day 1 adult worms that have been on the RNAi for two generations (except for *sams-1*, see above). A Direct-zol RNA miniprep kit (Zymo) was used for RNA extraction, and cDNA was generated using the Biorad iScript cDNA Synthesis Kit. Expression levels were measured with the SYBER Green (Bio-Rad) quantitative reverse transcription PCR system, and mRNA levels were normalized to the housekeeping genes *cdc-42* and *Y45FD10.4*. Primers for RNAi validation were designed to span the 3′ untranslated region of each transcript to prevent amplification of the bacterially produced RNAi. Gene expression was calculated using standard curves.

### Enzymatic activity assay

The oxygenation activity of FMO-2 was assessed using a method described previously (*24*, *101*). In brief, the consumption of reduced form of nicotinamide adenine dinucleotide phosphate (NADPH) was monitored spectrophotometrically at 340 nm, using the molar extinction coefficient of 6.22 mM^−1^ cm^−1^ to determine substrate oxygenation. The assay buffer contained 25 mM sodium phosphate (pH 8.5), 0.5 mM diethylenetriaminepentaacetic acid (DETAPAC), 0.5 mM NADPH, 0.04 μM FMO-2, and excess flavin adenine dinucleotide (FAD). For tryptophan, the final concentrations were 100, 250, 500, and 750 μM and 1, 2.5, 5, 7.5, and 10 mM. For methimazole, the final concentrations were 100, 300, and 600 μM, along with 1, 3, 5, 7, 10, and 30 mM. To assess NADPH oxidation rates by FMO, NADPH concentrations of 10, 30, 100, 300, 500, and 700 μM, as well as 1 and 1.5 mM, were used. All experiments were performed at 30°C with shaking. Kinetic parameters (e.g., *K*_cat_ and *K*_m_) were determined by fitting the rate of turnover versus substrate concentration to the Michaelis-Menten equation, using GraphPad Prism (version 9.1.0). FMO-2 protein was purchased from GenScript, whereas NADPH (10107824001), FAD (F6625), methimazole, l-tryptophan (T0254), and other substrates were sourced from Sigma-Aldrich (St. Louis, MO). DETAPAC (AC114322500) and sodium phosphate buffer (S374-500) were obtained from Fisher (Waltham, MA).

### Serotonin immobilization assay

The serotonin immobilization assay was performed as previously described by Ranganathan *et al.* (*68*). Briefly, 20 to 30 worms were washed off standard NGM plates at day 1 of adulthood and rinsed twice with M9 to remove any residual bacteria. Animals were then placed in 200 μl of 30 mM serotonin solution (serotonin creatine sulfate salt in M9 buffer). Videos were recorded for 20 min after worms were placed in the serotonin solution, and an experimenter blinded to the condition in each video scored the number of mobile and immobile worms at 1, 2, 4, 5, 7.5, 10, 12.5, 15, 17.5, and 20 min. Worms were considered immobile if they did not swim for 5 s. Data were plotted by R version 4.3.1 and Adobe Illustrator 2022.

### Life-span measurements

Life-span assays were performed as previously described (*62*). In short, 10 to 15 gravid adults were transferred to NGM for a 3-hour timed egg-lay and then removed. Once their progeny reached day 1 of adulthood, 60 to 80 worms were transferred to NGM plates with 33 μl of 150 mM fluorodeoxyuridine (FUdR) and 100 μl of ampicillin (AMP; 50 mg/ml) per 100 ml of NGM. The FUdR prevents development of progeny, and the AMP prevents bacterial growth. NGM + FUdR + AMP plates were seeded with concentrated bacteria (5x for OP50-fed life spans), and at least two plates per strain per condition were used for each life-span replicate. Animals were removed from the experiment and counted as dead when they did not move in response to a gentle touch from a platinum wire pick under a dissection microscope. Life spans were scored at least three times per week until all animals were dead. A “fence” of 75 μl of 100 mM palmitic acid (Sigma-Aldrich) dissolved in 100% ethanol was also applied to the edge of each life-span plate to prevent fleeing. Data were plotted by R version 4.3.1 and Adobe Illustrator 2022.

### RNAi life spans

RNAi life-span assays were performed like other life spans except for the initial timed egg-lay and the food concentrations. To ensure maximum knockdown on RNAi, the first generation of worms was subjected to a timed egg-lay on RNAi for 3 to 4 hours. The progeny from this timed egg-lay was left on RNAi plates to develop into gravid adults and was then used for a second timed egg-lay under the same RNAi conditions. Progeny from the second-generation timed egg-lay was then used for the life-span assay. RNAi bacteria (HT115) is AMP resistant and can grow slowly on AMP life-span plates during the assay. To maintain equivalent amounts of bacterial availability over the course of the life span, RNAi life-span plates are seeded with 2x concentrated bacteria, from a starting OD_600_ of 3.0.

### Fluorescence slide microscopy

Fluorescent images in this study were taken using a Leica M165F fluorescence microscope running Leica Application Suite X (LASX) software. At least 15 worms per condition were imaged at ≥70x magnification. For representative images displayed in the figures, worms were paralyzed in 0.5 M sodium azide (NaN_3_) and imaged once residual liquid evaporated and the worms clumped together. Paralyzed worms were then gently rearranged, so the heads and tails faced the same orientation. For quantification of these images, individual worms were gently pushed apart until they were no longer touching one another. For each worm, an experimenter blinded to the condition traced and measured the mean fluorescence of the worm’s head and the cell body of each individual serotonergic neuron in ImageJ bundled with 64-bit Java 1.8.0. The mean fluorescence intensity of the background was subtracted from the mean fluorescence measurements. Data were plotted by R version 4.3.1 and Adobe Illustrator 2022.

### Targeted metabolomics

#### C. elegans collection for metabolomics

For metabolomics analysis, ~2000 worms per sample were washed off agar plates in ~10 ml of media and transferred to 15-ml conical-bottom polypropylene tubes. Worms were centrifuged gently (1 min at 1 rcf) to produce a diffuse pellet. The supernatant was removed, and worms were resuspended in 5 ml of deionized water to rinse and remove residual media. The suspended worms were centrifuged again, and the supernatant was removed, followed by a second wash with 5 ml of deionized water. The supernatant was removed again after the second rinse, leaving a volume of ~100 μl of an aqueous suspension of worms at the bottom of the tube. This suspension was flash-frozen by immersing the tube in liquid nitrogen, and the tubes were stored at −80°C until metabolite extraction.

#### Metabolite extraction

The above-described frozen pellets were extracted directly in the 15-ml tubes using 300 μl of ice-cold acetonitrile containing 5 μM D5-tryptophan, which served as an internal standard, resulting in a 3:1 acetonitrile:water solvent ratio. Immediately following the addition of acetonitrile, the sample was disrupted using 30 pulses of a Branson 450 ultrasonic probe set at 40% duty cycle and power level 4. The sample was vortexed after 15 pulses to ensure any solid material on the sides of the tube was returned to the suspension. All samples were allowed to remain on wet ice for at least 30 min following sonication to ensure complete metabolite extraction. The tubes were then centrifuged at 5000*g* for 10 min at 4°C to pellet cell debris. The supernatant was split into two aliquots, which were chemically derivatized before liquid chromatography–mass spectrometry (LC-MS) analysis as described below.

#### *Benzoyl chloride derivatization for nitrogen-containing tryptophan intermediates (e.g., serotonin*)

Derivatization was performed according to a previously described protocol (*102*). Forty microliters of the *C. elegans* supernatant extract from each sample was transferred to labeled glass autosampler vials fitted with 350-μl flat-bottom glass inserts. Twenty microliters of 100 mM sodium carbonate in water was added, and vials were vortexed. Then, 20 μl of 2% (v/v) benzoyl chloride (BzCl) in acetonitrile was added to the vials and vortexed, after which 20 μl of 1% sulfuric acid (v/v) in 8:2 water:acetonitrile was added and vortexed. Last, the samples were diluted using 50 μl of LC-MS grade water and vortexed, after which they were analyzed by LC-MS as described below.

#### 3-nitrophenylhydrazine derivatization of organic acids

Compounds containing organic acids were derivatized using a modified version of a previously described protocol (*103*)*.* A second 40-μl aliquot of the *C. elegans* extract supernatant was transferred to another set of labeled glass autosampler vials with flat-bottom inserts. To these vials were added 10 μl of 200 mM 3-nitrophenylhydrazine (3-NPH) dissolved in in 1:1 acetonitrile:water, followed by 10 μl of 120 mM 1-ethyl-3-(3-dimethylaminopropyl)carbodiimide dissolved in 47:47:1 acetonitrile:water:pyridine. The vials were capped, vortexed, and warmed to 40°C for 30 min to complete derivatization. To quench the reaction,10 μl of 200 mM formic acid and 10 μl of 120 mM 2-mercaptoethanol were added and vortexed to mix. The vials were recapped and warmed to 40°C for 10 min. An additional 20 μl of LC-MS grade water were added and vortexed, after which the samples were analyzed by LC-MS.

#### LC-MS analysis

LC-MS analysis of both sets of derivatized samples was performed using an Orbitrap ID-X mass spectrometer coupled to a Vanquish Horizon UPLC system. All LC and MS parameters were identical for BzCl- and 3-NPH–derivatized samples, except that BzCl samples were analyzed using positive ion mode electrospray and 3-NPH samples were analyzed in negative ion mode. The column used was a Waters HSS T3 (100 mm by 2.1 mm in inner diameter with 1.8-μm particle size) and was equipped with a matched Vanguard guard column. Mobile phase A was 0.1% formic acid in water, and mobile phase B was 0.025% formic acid in methanol. The gradient consisted of a linear ramp from 0 to 100% B over 10 min, a 6-min hold at 100% B, after which the mobile phase returned immediately to 0% B and was held for 3 min to reequilibrate the column before the next run. The column temperature was set at 55°C, the flow rate was 0.45 ml/min, and the injection volume was 10 μl. MS source parameters were as follows: spray voltage, 3500-V positive/2500-V negative; sheath gas, 50; aux gas, 10; sweep gas, 1; ion transfer tube temperature, 325°C; and vaporizer temperature, 350°C. MS scan parameters were as follows: scan mode, MS1 full scan; orbitrap resolution, 120,000; quadrupole isolation, enabled; scan range, mass/charge ratio (*m/z*) 100 to 1000; RF lens, 50%; automatic gain control target, standard; maximum injection time mode, auto; data type, centroid; and Easy-IC (internal calibration), enabled. Data were analyzed for targeted tryptophan intermediates using Skyline, which was configured with expected *m/z* and retention time (RT) of compounds of interest determined by analysis of authentic standards derivatized using the protocols described above. *m/z* and RT parameters, including the used for targeted intermediates, are displayed below. To account for environmental differences in cell mass across biological replicates, the peak height for each compound of interest was normalized to the peak height of the WT control from the same collection date when comparing abundance between groups. Details for each compound measured appear below:

Serotonin: Derivatization: +2(Bz); formula: C_24_H_21_N_2_O_3_; *m/z* (M+H) (Bz); 385.1547; RT (min): 8.31

Kynurenic acid: Derivatization: +(3-NPH); formula: C_16_H_11_N_4_O_4_; (M−H) (3-NPH); 323.0786; RT (min): 6.39

Quinolinic acid: Derivatization: +2(3-NPH); formula: C_19_H_14_N_7_O_6_; (M−H) (3-NPH); 436.101105; RT (min): 6.35

### Statistical analysis

The height of all bar plots represents the mean of the condition, and error bars indicate the SEM. For all comparisons of >2 conditions, an ANOVA and Tukey post hoc tests (two-tailed, unpaired) were run to determine interactions between variables and derive statistical comparisons between each condition. *P* values are *P < 0.05, **P < 0.01, ***P < 0.001, and ****P < 0.0001. For life-span assays, the statistical groupwise and pairwise comparisons across survivorship curves were performed using the survfit survival analysis function in the survival package in R (*104*). *P* values comparing two survival curves were acquired using the log-rank analysis. *P* values indicating an interaction between multiple experimental variables and across >2 survival curves were performed using the survivalMPL package in R to perform a Cox regression analysis between covariates of interest. Tests done with data from multiple biological variables were adjusted with a Bonferroni *P* value correction. *P* values are *P < 0.05, **P < 0.01, ***P < 0.001, and ****P < 0.0001.
