# Supplementary material for: Metabolic regulation of behavior by the intestinal enzyme FMO-2
Source: Sci Adv. 2025 Oct 24;11(43):eadx3018. doi: 10.1126/sciadv.adx3018 (PMC12551704; doi:10.1126/sciadv.adx3018)
Supplement: Supplementary file 1 — Figs. S1 to S7 Tables S1 and S2 Legends for data S1 to S13 [file sciadv.adx3018_sm.pdf]

Science Advances
AAAS

Supplementary Materials for

Metabolic regulation of behavior by the intestinal enzyme FMO-2

Elizabeth S. Kitto *et al.*

Corresponding author: Scott F. Leiser, leiser@umich.edu

Sci. Adv. **11**, eadx3018 (2025)
DOI: 10.1126/sciadv.adx3018

The PDF file includes:

Figs. S1 to S7
Tables S1 and S2
Legends for data S1 to S13

Other Supplementary Material for this manuscript includes the following:

Data S1 to S13

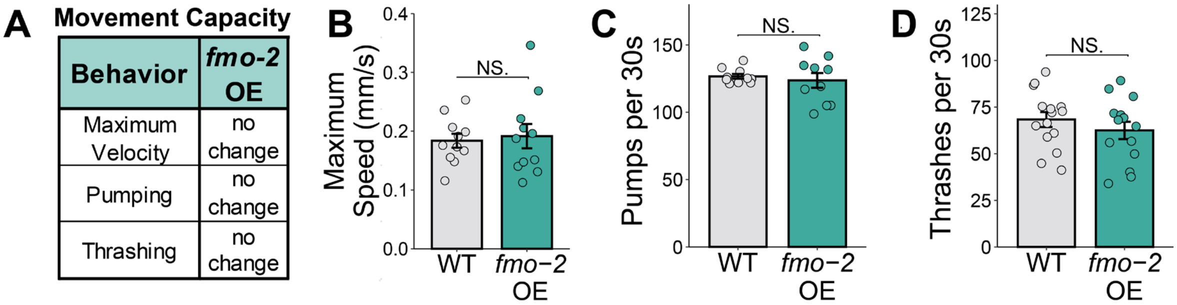

Fig. S1. ***fmo-2*** **overexpressing animals have normal movement capacity during young adulthood.**

(**A**) A summary of the movement capacity of *fmo-2* overexpressing animals relative to WT. (**B**) Quantification of the maximum velocity of WT and *fmo-2* overexpressing worms over a 3-minute video recording. $N \geq 11$ worms per condition. (**C-D**) Quantification of the pumping (**C**) and thrashing (**D**) rates of WT and *fmo-2* overexpressing animals. $N \geq 10$ (**C**) or $N \geq 15$ (**D**) worms per condition. In all bar plots, the top of the bar represents the mean of the population and error bars indicate standard error of the mean (SEM). In all panels, NS. = $p > 0.05$. Significance for all panels is from a one-way ANOVA and post-hoc Tukey HSD test (unpaired, two-tailed). All panels show one representative replicate. Raw data from all three replicates can be found in supplemental data files.

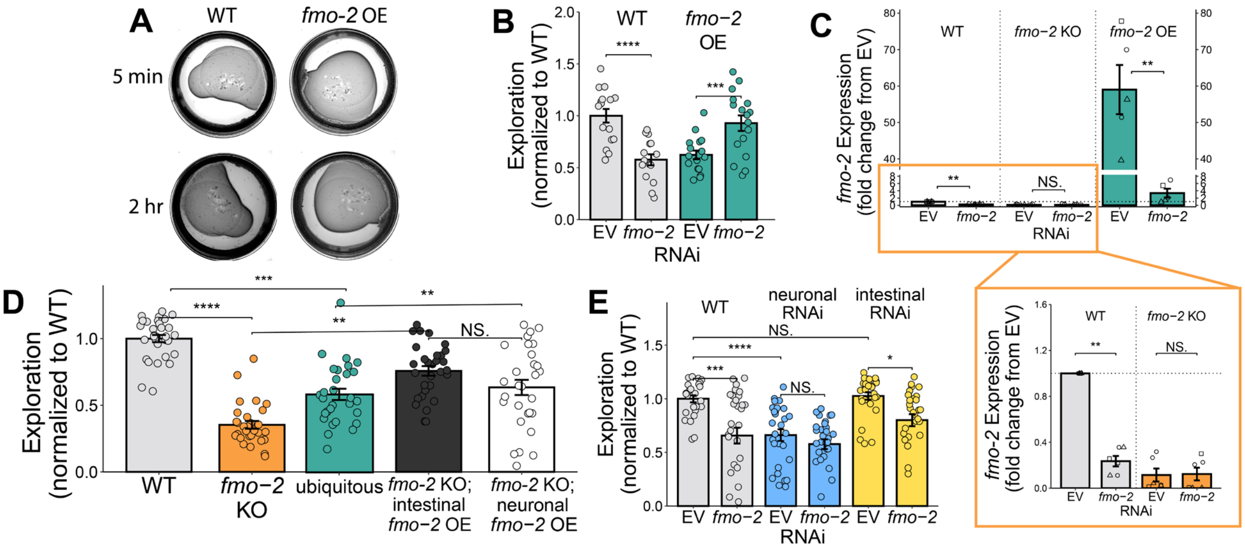

Fig. S2. The effects of *fmo-2* expression on exploratory behavior persist through mid-adulthood and both neural and intestinal *fmo-2* expression contribute to changes in exploratory behavior.

(**A**) Representative images of pathogenic lawn occupancy, quantified in (**Fig. 1F**). (**B**) Exploration of WT and *fmo-2* overexpressing animals on empty vector (EV) or *fmo-2* RNAi. $N \geq$ 16 worms per condition. (**C**) Gene expression of *fmo-2* in WT, *fmo-2* KO, and *fmo-2* OE worms raised on *fmo-2* RNAi compared to control worms raised on empty vector (EV) RNAi for two generations. $N \geq$ 200 worms per replicate, or 800 worms per condition. WT and *fmo-2* KO worms are also plotted on a different y-axis scale to allow for clear visualization of each condition. (**D**) Exploration of WT, *fmo-2* knockout, ubiquitous *fmo-2* overexpressors *(eft-3p::fmo-2)*, intestinal only *fmo-2* rescue (*fmo-2(ok2147); elt-2p::fmo-2*), and neuronal only *fmo-2* rescue (*fmo-2(ok2147); rab-3p::fmo-2*) strains. $N \geq$ 29 worms per condition. (**E**) Exploratory behavior of WT, neuronal specific RNAi uptake (*sid-1(qt9); rgef-1p::sid-1*), and intestinal specific RNAi uptake (*sid-1(qt9); vha-6p::sid-1*) strains on empty vector (EV) and *fmo-2* RNAi. $N \geq$ 23 worms per condition. In all bar plots, the top of the bar represents the mean of the population and error bars indicate standard error of the mean (SEM). In all panels, NS. = $p >$ 0.05, * = $p < 0.05$, ** = $p < 0.01$, *** = $p < 0.001$, and **** = $p < 0.0001$. Significance for all panels is from a one-way (panel D) or two-way (panels B,C,E) ANOVA and post-hoc Tukey HSD test (unpaired, two-tailed). All panels show one representative replicate except for panel C, which includes all replicates. Raw data from three individual replicates for each experiment can be found in supplemental data files.

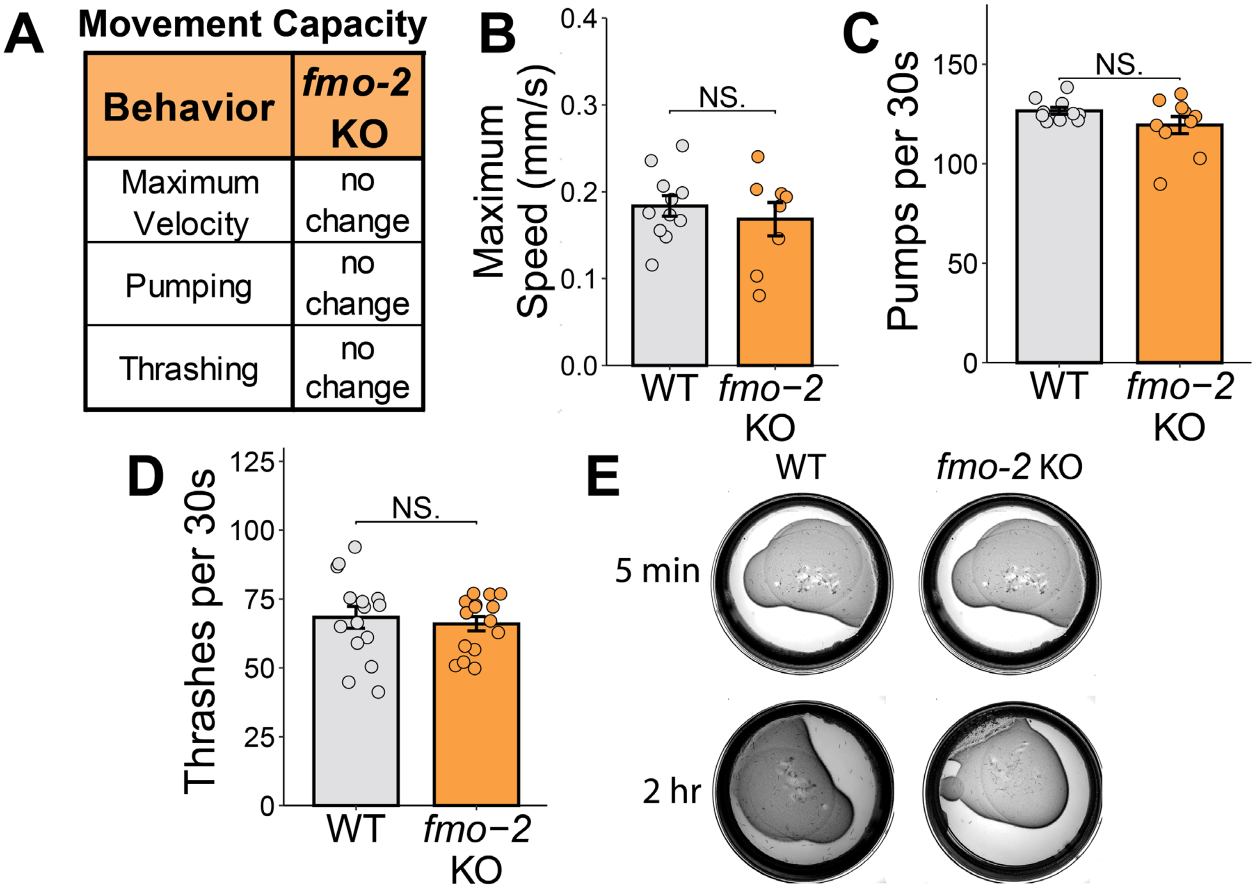

Fig. S3. ***fmo-2*** **knockout animals have normal movement capacity during young adulthood.** (**A**) A summary of the movement capacity of *fmo-2* knockout animals relative to WT. (**B**) Quantification of the maximum velocity of WT and *fmo-2* knockout worms over a 3-minute video recording. $N \geq 8$ worms per condition. (**C-D**) Quantification of the pumping (**C**) and thrashing (**D**) rates of WT and *fmo-2* knockout animals. $N \geq 10$ (**C**) or $N \geq 15$ (**D**) worms per condition. (**E**) Representative images of lawn-fleeing data quantified in Figure 2F. In all bar plots, the top of the bar represents the mean of the population and error bars indicate standard error of the mean (SEM). In all panels, NS. = $p > 0.05$. Significance for all panels is from a one-way ANOVA and post-hoc Tukey HSD test (unpaired, two-tailed). All panels show one representative replicate. Raw data from all three replicates can be found in supplemental data files.

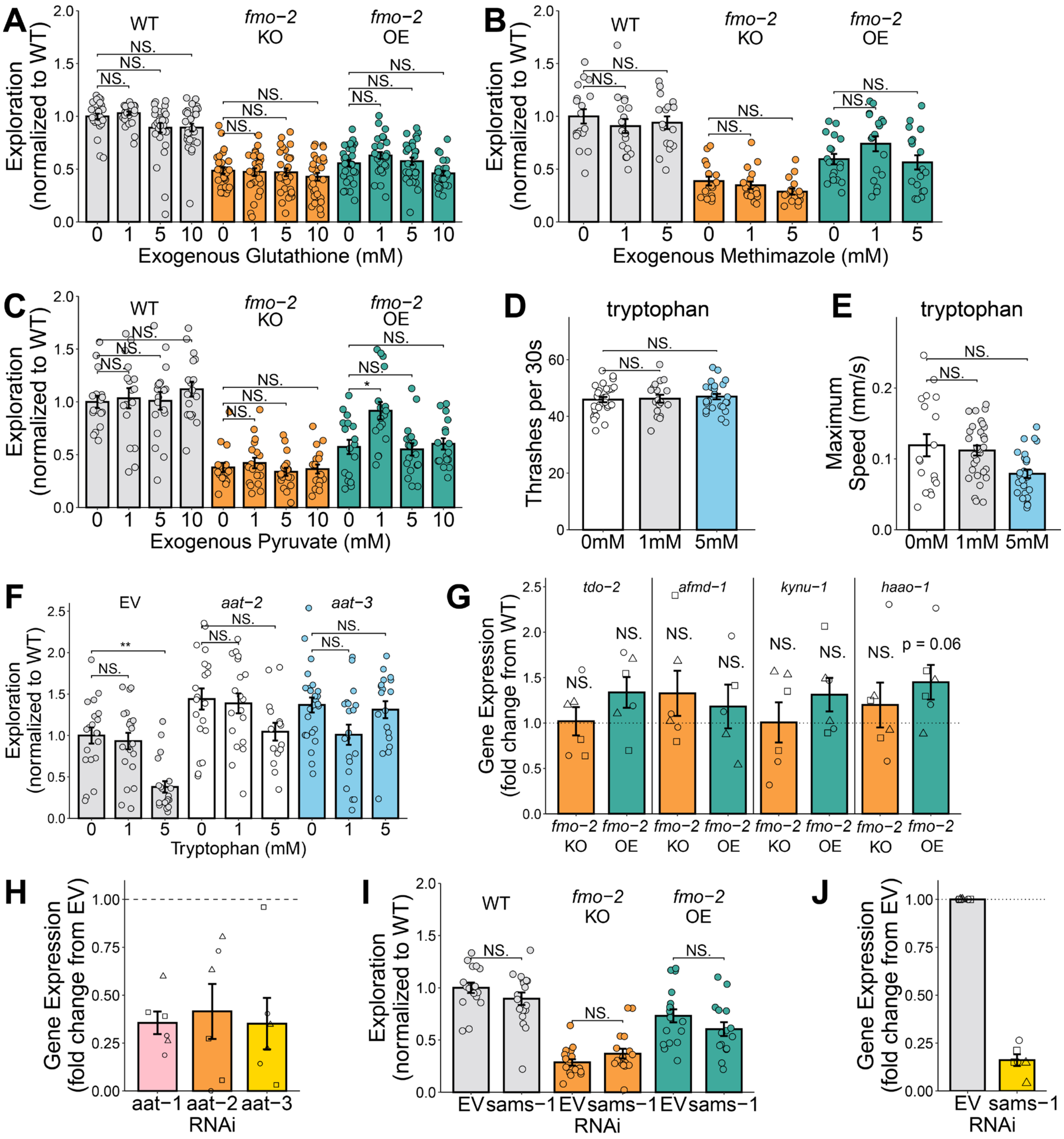

Fig. S4. Supplementation with glutathione, methimazole, and pyruvate do not modify exploration, knocking down *aat-2* and *aat-3* blocks tryptophan-mediated decreases in exploration, and *sams-1* does not interact with *fmo-2* to modify exploration.
(**A**-**C**) Exploration of WT, *fmo-2* knockout, and *fmo-2* overexpressing worms after supplementation from egg with glutathione (**A**), methimazole (**B**), and pyruvate (**C**). $N \geq 29$ (**A**), ≥ 21 (**B**), and ≥ 20 (**C**) worms per condition. (**D-E**) Quantification of the thrashing rate (**D**) and maximum velocity (**E**) of WT worms on control or tryptophan-supplemented plates. $N \geq 22$ (**C**) or $N \geq 18$ (**D**) worms per condition. (**F**) Exploration of WT worms raised on empty vector (EV), *aat-2*, and *aat-3* RNAi on 0, 1, and 5 mM tryptophan from egg. $N \geq 25$ worms per condition. (**G**) Gene expression of *tdo-2, afmd-1, kynu-1*, and *haao-1* in *fmo-2* KO and OE worms compared to WT controls. $N \geq 200$ worms per replicate, or 1,200 worms per condition. (**H**) Gene expression

of *aat-1*, *aat-2*, and *aat-3* in worms raised on *aat-1, aat-2,* and *aat-3* RNAi compared to control worms raised on empty vector (EV) RNAi for two generations. $N \geq 200$ worms per replicate, or 1,200 worms per condition. (**I**) Exploration of WT, *fmo-2* knockout, and *fmo-2* overexpressing worms on empty vector (EV) or *sams-1* RNAi. $N \geq 17$ worms per condition. (**J**) Gene expression of *sams-1* in worms raised on *sams-1* RNAi compared to control worms raised on empty vector (EV) RNAi from L3 stage. $N \geq 200$ worms per replicate, or 1,200 worms per condition. In all bar plots, the top of the bar represents the mean of the population and error bars indicate standard error of the mean (SEM). In all panels, NS. = $p > 0.05$, * = $p < 0.05$, ** = $p < 0.01$, *** = $p < 0.001$, and **** = $p < 0.0001$. Significance for all panels is from a two-way ANOVA and post-hoc Tukey HSD test (unpaired, two-tailed). All panels except G-H and J show one representative replicate. Raw data from all three replicates can be found in supplemental data files. Panels G-H and J show all replicates plotted together.

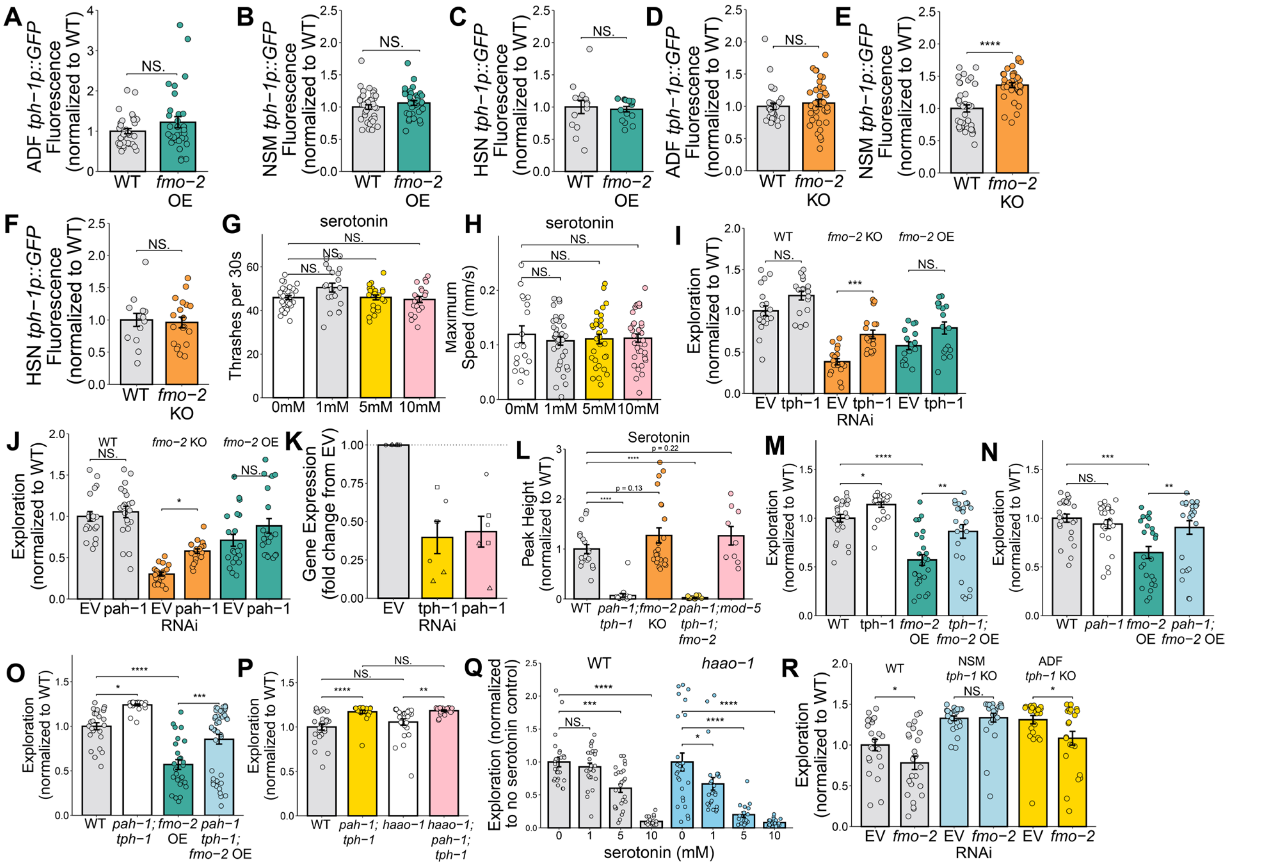

Fig. S5. Knocking out *fmo-2* increases *tph-1p::GFP* fluorescence in the NSM neurons and knocking down *tph-1* or *pah-1* partially rescues the exploration of the *fmo-2* knockout. (**A-C**) Quantification of the mean fluorescence of *tph-1p::GFP* in a WT and *fmo-2* overexpressing background in the (**A**) ADF, (**B**) NSM, and (**C**) HSN neurons. $N \geq 29$ (**A**), ≥ 28 (**B**), and ≥ 22 (**C**), neurons per condition. (**D-F**) Quantification of the mean fluorescence of *tph-1p::GFP* in a WT and *fmo-2* knockout background in the (**D**) ADF, (**E**) NSM, and (**F**) HSN neurons. $N \geq 33$ (**D**), ≥ 36 (**E**), and ≥ 19 (**F**), neurons per condition. (**G-H**) Quantification of the thrashing rate (**G**) and maximum velocity (**H**) of WT worms on control or serotonin-supplemented plates. $N \geq 20$ (**G**) or $N \geq 18$ (**H**) worms per condition. (**I**) Exploration of WT, *fmo-2* knockout, and *fmo-2* overexpressing worms on empty vector (EV) or *tph-1* RNAi. $N \geq 17$ worms per condition. (**J**) Exploration of WT, *fmo-2* knockout, and *fmo-2* overexpressing worms on empty vector (EV) or *pah-1* RNAi. $N \geq 24$ worms per condition. (**K**) Gene expression of *tph-1* and *pah-1* in worms raised on *tph-1* and *pah-1* compared to control worms raised on empty vector (EV) RNAi for two generations. $N \geq 200$ worms per replicate, or 1,200 worms per condition. (**L**) Serotonin abundance in WT, *pah-1; tph-1*, *fmo-2 KO*, *pah-1; tph-1; fmo-2*, and *mod-5* animals. $N \geq 20{,}000$ worms per condition. Each datapoint represents one biological sample of ~2,000 worms. (**M**) Exploration of WT, *fmo-2* overexpressing, *tph-1* knockout, and *tph-1; fmo-2* overexpressing worms. $N \geq 24$ worms per condition. (**N**) Exploration of WT, *fmo-2* overexpressing, *pah-1* knockout, and *pah-1; fmo-2* overexpressing worms. $N \geq 22$ worms per condition. (**O**) Exploration of WT, *fmo-2* overexpressing, *tph-1; pah-1* double knockout, and *tph-1; pah-1; fmo-2* overexpressing worms. $N \geq 25$ worms per condition. (**P**) Exploration of WT, *haao-1*, *pah-1; tph-1,* and *haao-1; pah-1; tph-1* worms. $N \geq 23$ worms per condition. (**Q**) Exploration of WT and *haao-1* knockout worms on 0-10mM serotonin. $N \geq 23$ worms per

condition. (**R**) Exploration of WT, NSM *tph-1* KO, and ADF *tph-1* KO worms on empty vector (EV) or *fmo-2* RNAi for 2 generations. $N \geq 23$ worms per condition. In all bar plots, the top of the bar represents the mean of the population and error bars indicate standard error of the mean (SEM). In all panels, NS. = $p > 0.05$, * = $p < 0.05$, ** = $p < 0.01$, *** = $p < 0.001$, and **** = $p < 0.0001$. Significance in panels I, J, and M-O is from a two-way ANOVA and post-hoc Tukey HSD test (unpaired, two-tailed). Significance for panels A-F is from a Wilcox test (two-tailed, unpaired). All panels show one representative replicate except for panels K and L, which show all replicates together. Raw data from individual three replicates can be found in supplemental data files.

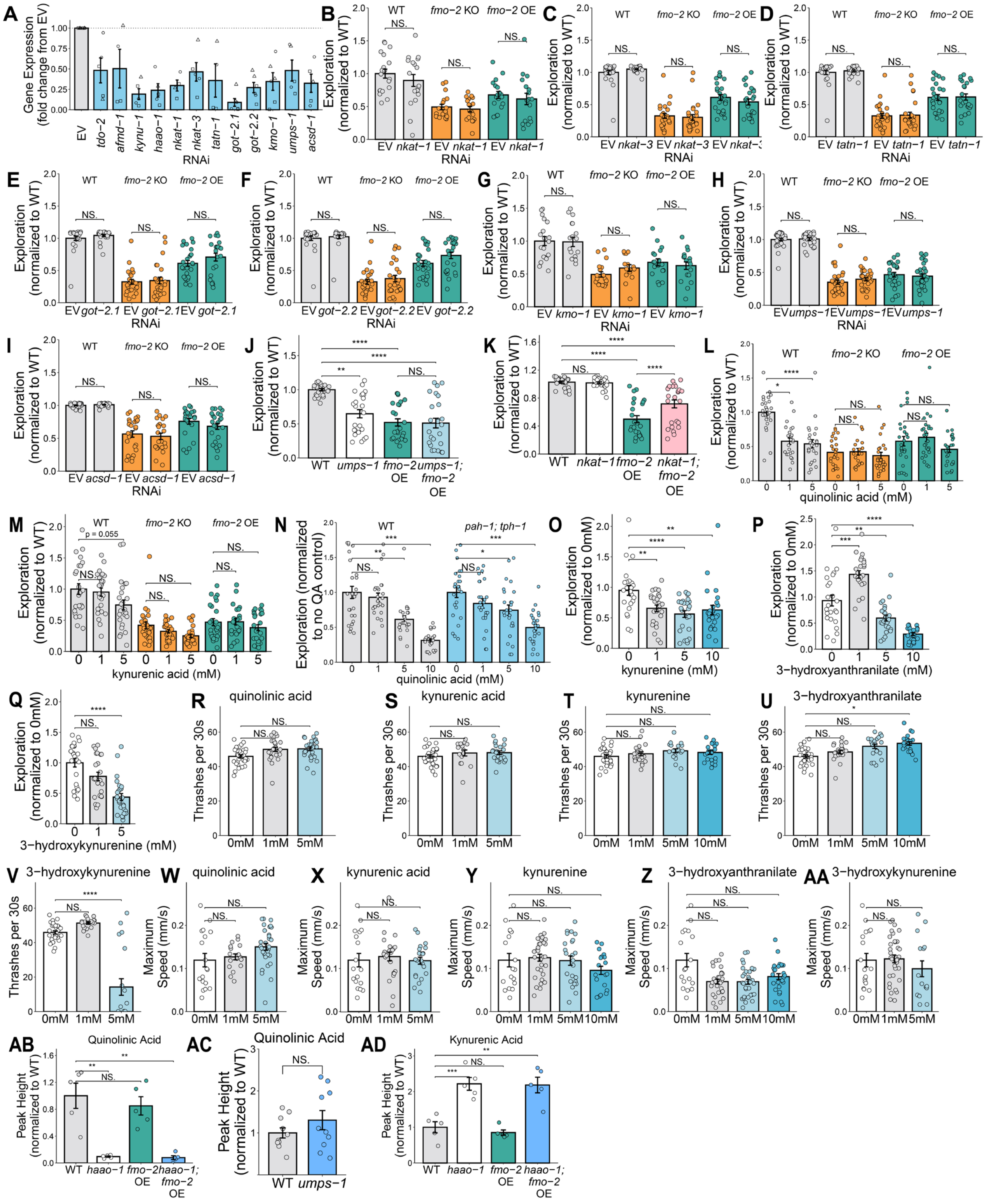

Fig. S6. Additional data from the tryptophan metabolism RNAi knockdown and knockout screen.

(**A**) Gene expression of *tdo-2, afmd-1, kynu-1, haao-1, nkat-1, nkat-3, tatn1, got-2.1, got-2.2, kmo-1, umps-1,* and *acsd-1* in worms raised on each RNAi compared to control worms raised on empty vector (EV) RNAi for two generations. $N \geq 200$ worms per replicate, or 800 worms per

condition. (**B-I**) Exploration of WT, *fmo-2* knockout, and *fmo-2* overexpressor worms on empty vector (EV) or (**B**) *nkat-1*, (**C**) *nkat-3*, (**D**) *tatn-1*, (**E**) *got-2.1*, (**F**) *got-2.2*, (**G**) *kmo-1*, (**H**) *umps-1*, and (**I**) *ascd-1* RNAi. $N \geq 17$ (**B**), ≥ 24 (**C**), ≥ 23 (**D**), ≥ 22 (**E**), ≥ 25 (**F**), ≥ 16 (**G**), ≥ 27 (**H**), and ≥ 23 (**I**) worms per condition. (**J**) Exploration of WT, *umps-1(zu456)*, *fmo-2* overexpressor, and *umps-1(zu456); fmo-2* overexpressor worms. $N \geq 24$ worms per condition. (**K**) Exploration of WT, *nkat-1(ok585)*, *fmo-2* overexpressor, and *nkat-1(ok585); fmo-2* overexpressor worms. $N \geq 25$ worms per condition. (**L-M**) Exploration of WT, *fmo-2* KO, and *fmo-2* OE on 0, 1, and 5 mM exogenous quinolinic acid (**L**) or kynurenic acid (**M**) from egg. $N \geq 24$ (**L**) and $N \geq 23$ (**M**) worms per condition. (**N**) Exploration of WT and *pah-1; tph-1* worms with 0-10 mM of exogenous quinolinic acid. $N \geq 24$ worms per condition. (**O-P**) Exploration of WT worms on 0, 1, 5, or 10 mM kynurenine (**O**) or 3-hydroxyanthralinate (**P**) from egg. $N \geq 25$ worms per condition. (**Q**) Exploration of WT worms on 0, 1, or 5 mM 3-hydroxykynurenine from egg. $N \geq 25$ worms per condition. (**R-V**) Thrashing of WT worms supplemented with 0-10mM quinolinic acid (**R**), kynurenic acid (**S**), kynurenine (**T**), 3-hydroxyanthralinate (**U**), or 3-hydroxykynurenine (**V**) from egg. $N \geq 24$ (**R**), $N \geq 15$ (**S**), $N \geq 19$ (**T**), $N \geq 18$ (**U**), and $N \geq 21$ (**V**) worms per condition. (**W-AA**) Maximum velocity over a 3-minute video of WT worms supplemented with 0-10 mM quinolinic acid (**W**), kynurenic acid (**X**), kynurenine (**Y**), 3-hydroxyanthralinate (**Z**), or 3-hydroxykynurenine (**AA**) from egg. $N \geq 23$ (**W**), $N \geq 23$ (**X**), $N \geq 29$ (**Y**), $N \geq 35$ (**Z**), and $N \geq 16$ (**AA**) worms per condition. (**AB**) Quinolinic acid abundance in WT, *haao-1*, *fmo-2* OE, and *haao-1; fmo-2* OE animals. $N \geq 10{,}000$ worms per condition. (**AC**) Quinolinic acid abundance in WT and *umps-1* animals. $N \geq 20{,}000$ worms per condition. (**AD**) Kynurenic acid abundance in WT, *haao-1*, *fmo-2* OE, and *haao-1; fmo-2* OE animals. $N \geq 10{,}000$ worms per condition. In all bar plots, the top of the bar represents the mean of the population and error bars indicate standard error of the mean (SEM). In all panels, NS. = $p > 0.05$, * = $p < 0.05$, ** = $p < 0.01$, *** = $p < 0.001$, and **** = $p < 0.0001$. Significance in all panels is from a one-way (panels K-J) or two-way (panels B-I, L-Z) ANOVA and post-hoc Tukey HSD test (unpaired, two-tailed). All panels show one representative replicate except for panels A and W-AD, which show all replicates together. Each datapoint in AB-AD represents one biological replicate of ~2,000 animals. Raw data from individual three replicates can be found in supplemental data files.

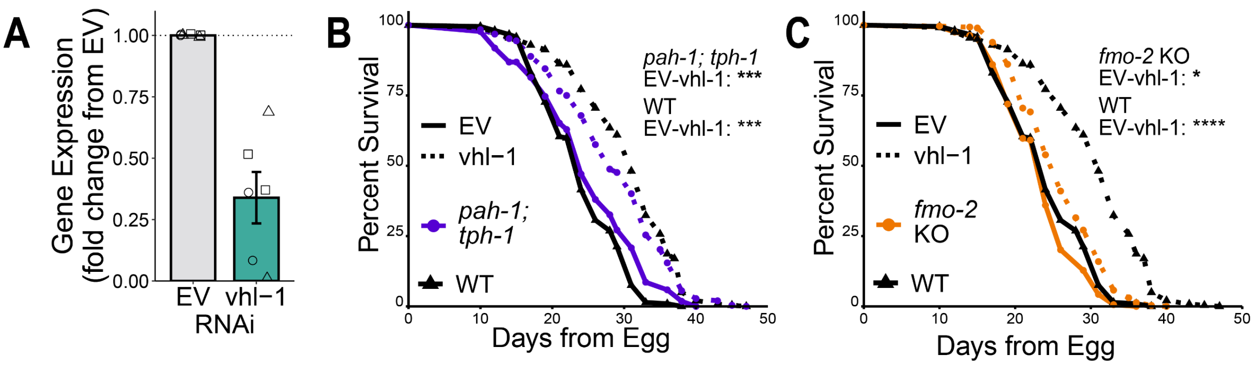

Fig. S7. ***vhl-1*** **RNAi extends lifespan in WT and** ***pah-1; tph-1*** **but this effect is partially blocked by** ***fmo-2*** **knockout.**
(**A**) Gene expression of *vhl-1* in worms raised on *vhl-1* RNAi compared to control worms raised on empty vector (EV) RNAi for two generations. $N \geq 200$ worms per replicate, or 1,200 worms per condition. (**B**) Survival curve of WT and *pah-1(syb3601); tph-1(mg280)* worms on empty vector (EV) or *vhl-1* RNAi. $N \geq 229$ worms per condition. (**C**) Survival curve of WT and *fmo-2(ok2147)* worms on empty vector (EV) or *vhl-1* RNAi. $N \geq 197$ worms per condition. In all panels, NS. = $p > 0.05$, * = $p < 0.05$, ** = $p < 0.01$, *** = $p < 0.001$, and **** = $p < 0.0001$. Significance in panels B-C is from a log-rank test comparing median survival on EV to *vhl-1* RNAi for each genotype. All panels plot data from three replicates together. Statistics for these panels include a Bonferroni correction for multiple comparisons.

RNAi	Figure	Library
fmo-2	S2	Vidal
aat-1	2	Vidal
aat-2	2	Vidal
aat-3	2	Vidal
sams-1	S3	Vidal
tph-1	S4	Vidal
pah-1	S4	Vidal
tdo-2	4	Vidal
afmd-1	4	Vidal
kynu-1	4	Vidal
haao-1	4	Vidal
nkat-1	S5	Vidal
nkat-3	S5	Vidal
tatn-1	S5	Vidal
got-2.1	S5	Vidal
got-2.2	S5	Vidal
kmo-1	S5	Vidal
umps-1	S5	Vidal
acsd-1	S5	Vidal
vhl-1	5	Vidal

Table S1.
RNAi used in this work.

Strain Description	Strain	Source	Identifier	Outcross
WT	wild-type	CGC	N2	≥4
fmo-2 knockout	*fmo-2(ok2147)*	CGC	VC1668	≥4
fmo-2 overexpressor (ubiquitous)	*seaSi39 [(pCFJ448) (eft-3p::fmo-2 + H2B::GFP) + Cbr-unc-119(+)] I.*	CGC	KAE9	≥4
fmo-2 overexpressor (intestinal)	*[elt-2p::fmo-2 + SL2::GFP]*	this paper	SCL46	≥4
fmo-2 overexpressor (neuronal)	*[rab-3p::fmo-2 + SL2::GFP]*	this paper	SCL48	≥4
fmo-2 overexpressor (muscle)	*[unc-54p::fmo-2 + SL2::GFP]*	this paper		
intestine-specific *fmo-2* rescue	*fmo-2(ok2147); [elt-2p::fmo-2 + H2B::GFP]*	this paper		≥4
neuronal-specific *fmo-2* rescue	*fmo-2(ok2147); [rab-3p::fmo-2 + H2B::GFP]*	this paper		≥4
neuronal-only RNAi uptake	*sid-1(qt9); sqIs71 [rgef-1p::GFP + rgef-1p::sid-1]*	CGC	MAH677	≥4
intestinal-only RNAi uptake	*sid-1(qt9); alxIs9 [vha-6p::sid-1::SL2::GFP]*	CGC	MGH171	≥4
fmo-1 knockout	*fmo-1(ok405)*	CGC		≥4
fmo-1 overexpressor	*[eft-1p::fmo-1]*	this paper		≥4
fmo-4 knockout	*fmo-4(ok294)*	CGC		≥4
fmo-4 overexpressor	*[eft-3p::fmo-4]*	Tuckowski *et al.*, 2024 (*81*)		≥4
mod-5 knockout	*mod-5(n3314)*	CGC	MT9772	≥4
tph-1 transcriptional reporter	*zdIs13 [tph-1p::GFP] IV.*	CGC	SK4013	≥4
fmo-2 KO; *tph-1* transcriptional reporter	*zdIs13 [tph-1p::GFP] IV.; fmo-2(ok2147)*	this paper		≥4

fmo-2 OE; *tph-1* transcriptional reporter	*zdIs13 [tph-1p::GFP] IV.; seaSi39 [(pCFJ448) (eft-3p::fmo-2 + H2B::GFP) + Cbr-unc-119(+)] I.*	this paper		≥4
tph-1 knockout	*tph-1(mg280)*	CGC	MT15434	≥4
pah-1 knockout	*pah-1(syb3601)*	CGC	PHX3601	≥4
pah-1; tph-1	*tph-1(mg280); pah-1(syb3601)*	CGC	PHX3596	≥4
pah-1; fmo-2 knockout	*pah-1(syb3601); fmo-2(ok2147)*	this paper		≥4
tph-1; fmo-2 knockout	*tph-1(mg280); fmo-2(ok2147)*	this paper		≥4
fmo-2 KO; *pah-1; tph-1*	*tph-1(mg280); pah-1(syb3601); fmo-2(ok2147)*	this paper		≥4
afmd-1 knockout	*afmd-1(tm4547)*	CGC	OW477	≥4
kynu-1 knockout	*kynu-1(tm4924)*	CGC	OW454	≥4
haao-1 knockout	*haao-1(tm4627)*	CGC	OW479	≥4
afmd-1 knockout*;* *fmo-2* OE	*afmd-1(tm4547); seaSi39 [(pCFJ448) (eft-3p::fmo-2 + H2B::GFP) + Cbr-unc-119(+)] I.*	this paper		≥4
kynu-1 knockout*;* *fmo-2 OE*	*kynu-1(tm4924); seaSi39 [(pCFJ448) (eft-3p::fmo-2 + H2B::GFP) + Cbr-unc-119(+)] I.*	this paper		≥4
haao-1 knockout*;* *fmo-2 OE*	*haao-1(tm4627); seaSi39 [(pCFJ448) (eft-3p::fmo-2 + H2B::GFP) + Cbr-unc-119(+)] I.*	this paper		≥4
nmr-1; nmr-2 knockout	*nmr-1(ak4); nmr-2(ok3324)*	this paper		≥4
nmr-1; nmr-2 knockout*;* *fmo-2* OE	*nmr-1(ak4); nmr-2(ok3324); seaSi39 [(pCFJ448) (eft-3p::fmo-2 + H2B::GFP) + Cbr-unc-119(+)] I.*	this paper		≥4
umps-1 knockout	*umps-1(zu456)*	CGC	GH636	≥4
umps-1 knockout*;* *fmo-2 OE*	*umps-1(zu456); seaSi39 [(pCFJ448) (eft-3p::fmo-2 + H2B::GFP) + Cbr-unc-119(+)] I.*	this paper		≥4
nkat-1 knockout	*nkat-1(ok585)*	CGC	RB795	≥4

nkat-1 knockout*;* *fmo-2 OE*	*nkat-1(ok585); seaSi39 [(pCFJ448) (eft-3p::fmo-2 + H2B::GFP) + Cbr-unc-119(+)] I.*	this paper		≥4

Table S2.
C. elegans strains used in this work.

Supplementary Dataset. (separate zip folder)
Contains 13 files with all data from the paper. Details on each file are below:

Data S1. (separate file)
Data visualized in Figure 1.

Data S2. (separate file)
Data visualized in Figure 2.

Data S3. (separate file)
Data visualized in Figure 3.

Data S4. (separate file)
Data visualized in Figure 4.

Data S5. (separate file)
Data visualized in Figure 5.

Data S6. (separate file)
Data visualized in Figure 6.

Data S7. (separate file)
Data visualized in Figure S1.

Data S8. (separate file)
Data visualized in Figure S2.

Data S9. (separate file)
Data visualized in Figure S3.

Data S10. (separate file)
Data visualized in Figure S4.

Data S11. (separate file)
Data visualized in Figure S5.

Data S12. (separate file)
Data visualized in Figure S6.

Data S13. (separate file)
Data visualized in Figure S7.
